# Temporal and spatial tracking of ultrafast light-induced strain and polarization modulation in a ferroelectric thin film

**DOI:** 10.1126/sciadv.adi1160

**Published:** 2023-11-15

**Authors:** Ruizhe Gu, Vincent Juvé, Claire Laulhé, Houssny Bouyanfif, Gwenaëlle Vaudel, Aurélie Poirier, Brahim Dkhil, Philippe Hollander, Charles Paillard, Mads C. Weber, Daniel Sando, Stéphane Fusil, Vincent Garcia, Pascal Ruello

**Affiliations:** ^1^ Institut des Molécules et Matériaux du Mans, UMR 6283 CNRS, Le Mans Université, 72085 Le Mans, France.; ^2^ Synchrotron SOLEIL, L’Orme des Merisiers, Université Paris Saclay, 91190 Saint-Aubin, France.; ^3^ Université Paris-Saclay, CNRS UMR8502, Laboratoire de Physique des Solides, 91405, Orsay, France.; ^4^ Laboratoire de Physique de la Matière Condensée, UR2081, Université Jules Vernes Picardie, 80000 Amiens, France.; ^5^ Université Paris-Saclay, CentraleSupélec, CNRS-UMR8580, Laboratoire Structures, Propriétés et Modélisation des Solides, Gif-sur-Yvette, France.; ^6^ University of Arkansas, Physics Department, 825 W Dickson St., Fayetteville, AR 72701, USA.; ^7^ School of Materials Science and Engineering, UNSW Sydney, Kensington 2052, Australia.; ^8^ School of Physical and Chemical Sciences, University of Canterbury, Christchurch 8410 New Zealand.; ^9^ Unité Mixte de Physique CNRS, Thales, Université Paris-Saclay, Palaiseau 91767, France.

## Abstract

Ultrashort light pulses induce rapid deformations of crystalline lattices. In ferroelectrics, lattice deformations couple directly to the polarization, which opens the perspective to modulate the electric polarization on an ultrafast time scale. Here, we report on the temporal and spatial tracking of strain and polar modulation in a single-domain BiFeO_3_ thin film by ultrashort light pulses. To map the light-induced deformation of the BiFeO_3_ unit cell, we perform time-resolved optical reflectivity and time-resolved x-ray diffraction. We show that an optical femtosecond laser pulse generates not only longitudinal but also shear strains. The longitudinal strain peaks at a large amplitude of 0.6%. The access of both the longitudinal and shear strains enables to quantitatively reconstruct the ultrafast deformation of the unit cell and to infer the corresponding reorientation of the ferroelectric polarization direction in space and time. Our findings open new perspectives for ultrafast manipulation of strain-coupled ferroic orders.

## Teaser

X-ray and visible laser pulses enable tracking of ultrafast strain and polarization modulation in a ferroelectric film.

## INTRODUCTION

In piezoelectric and ferroelectric materials, the couplings between lattice distortions and the polar order are pivotal for key applications, such as smart electromechanical microelectronic components, spintronic memories, or advanced photonics (*1*–*8*). With the demand for high-rate data processing, the control of these couplings at ultrafast time scales, i.e., in the picosecond range, is desirable. One way to reach this ultrafast regime is the use of ultrashort laser pulses. For example, ultrashort light pulses have induced mechanical strains of up to 0.1% in ferroelectrics (*9*–*11*). These strain levels compare to commercial electrically driven piezoelectric sensors (*1*). Therefore, the ultrafast photoexcitation of piezoelectric and ferroelectric materials has triggered a large interest to control and modulate the strain (*9*–*16*) and to generate coherent acoustic (*17*–*20*) and optical phonons (*21*) with short light pulses. Even the switching of a ferroelectric polarization with short light pulses has recently been reported (*6*–*8*, *22*, *23*).

**Fig. 1.**
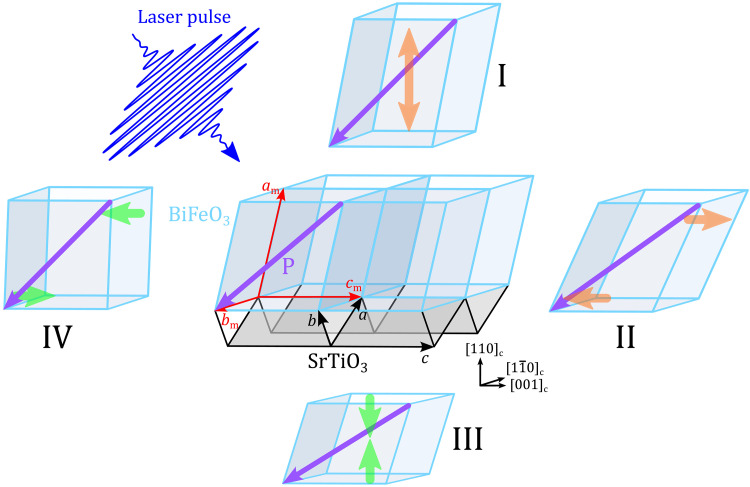
Light-induced deformation of a ferroelectric lattice. The central sketch shows the (100)_m_-oriented monoclinic BiFeO_3_ film (blue) on the (110)_c_-oriented SrTiO_3_ substrate (gray) at equilibrium. The purple arrow denotes the ferroelectric polarization (**P**). *a*_m_, *b*_m_, and *c*_m_ are the monoclinic axes of BiFeO_3_ and *a*, *b*, and *c* are the cubic axes of SrTiO_3_. The femtosecond laser pulse induces an out-of-plane longitudinal strain (sketches I and III) and an in-plane shear strain (sketches II and IV) in the ferroelectric lattice. The longitudinal and shear strains can be either positive (orange arrows, sketches I and II) or negative (green arrows, sketches III and IV). Positive (negative) longitudinal and shear strain values correspond to an out-of-plane expansion (contraction) and an increase (decrease) of the monoclinic distortion, respectively. The corresponding variation of the ferroelectric polarization direction is shown in purple for each strain component. The 12-nm buffer SrRuO_3_ layer is omitted for clarity.

Since the polar order and crystal lattice are intrinsically coupled by the piezoelectric effect, a full understanding of the structural distortions induced by light pulses is crucial for an insight into the light-driven manipulation of the polar order. To gain a full picture of ultrafast photo-induced distortions in ferroelectrics, access to the modulation of both the out-of-plane and in-plane components of the elastic distortions is essential. However, to date, several limitations exist. First of all, multidomain states, which are typical in ferroelectric materials, hamper this analysis, particularly in thin films with nanosize domains. The presence of various domains induces, indeed, an averaging effect on the photoinduced response, which precludes a straightforward description of the intrinsic interplay between light and lattice distortions. To obtain the clearest picture of these fundamental interactions, model systems are required, i.e., thin films consisting of a single ferroelectric domain. Second, most studies have been limited to the measurement of light-induced out-of-plane distortions precluding a full description of the light-induced lattice deformation (*9*, *10*, *12*, *14*–*16*). In the present work, we first of all overcome the limitation of the multidomain complexity by fabricating a high-quality epitaxial single-domain BiFeO_3_ film through strain and domain engineering (*24*–*27*). This archetypal system is a prerequisite for the understanding of the photo-induced response beyond bulk materials (*11*) or multidomains states (*10*, *15*, *28*). Second, we combine time-resolved optical and time-resolved x-ray diffraction (XRD) experiments to determine the complete light-induced ultrafast deformation of the unit cell in a BiFeO_3_ thin film (Fig. 1). It enables us to access both in-plane and out-of-plane lattice distortions of BiFeO_3_ films in the picosecond regime. Our results show that the ultrafast light excitation engenders a bipolar strain pulse with a leading compressive front, followed by a tensile tail for the longitudinal component. Concomitantly, positive and negative shear strain components are revealed in these leading front and tail, respectively. This temporal and spatial reconstruction of the longitudinal and shear strain has never been revealed before (*9*, *10*, *12*, *14*–*16*, *20*). For clarity, compressive (negative) and tensile (positive) components of the longitudinal (shear) strain are indicated by green and orange arrows in Fig. 1. The out-of-plane parameter reaches maximal compression and expansion of 0.1 and 0.6% respectively. Simultaneously, we observe a transient shear strain corresponding to the in-plane lattice distortion and of bipolar nature with a maximum amplitude of 0.04%. Our full temporal and spatial reconstruction of the transient lattice distortions points to successive reorientations of the ferroelectric polarization. Hence, ferroelectric polarization can be modulated on the picosecond time scale by a light-induced strain wave.

## RESULTS AND ANALYSIS

### Sample preparation and characterization

**Fig. 2.**
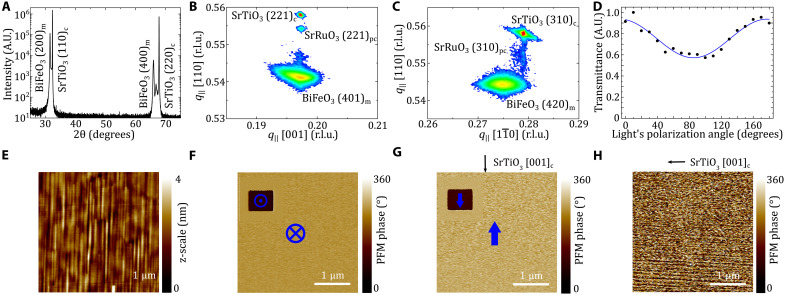
Single-domain BiFeO_3_ thin film on (110)_c_ SrTiO_3_. (**A**) θ-2θ XRD pattern of the single-domain monoclinic (100)_m_ BiFeO_3_ on the (110)_c_ SrTiO_3_ substrate. (**B** and **C**) Reciprocal space maps near the 221_c_ (B) and 310_c_ (C) substrate Bragg reflections. (**D**) Optical transmission measurement as a function of the probe light polarization rotation angle performed with a 400-nm laser. (**E** to **H**) PFM images revealing the presence of a single ferroelectric domain. The topography (E), the out-of-plane PFM phase (F), and in-plane PFM phase in two different directions [cantilever perpendicular (G) and parallel to (H) [001]_c_ SrTiO_3_, respectively] indicate that the polarization is pointing downward with an in-plane component antiparallel to [001]_c_ SrTiO_3_. The insets in (F) and (G) show the 180° switched polarization after applying a dc voltage of 7 V to the bottom electrode. r.l.u., reciprocal lattice unit; A.U., arbitrary units.

Figure 1 shows a sketch of our (100)_m_-oriented [i.e., (110)_pc_], 180-nm BiFeO_3_ film grown on (110)_c_ SrTiO_3_ with a 12-nm SrRuO_3_ electrode [m, monoclinic; c, cubic; pc, pseudocubic; see Methods and (*29*, *30*) for further information on the growth process]. From XRD measurements, we identify the (100)_m_ film orientation (Fig. 2A) and find the lattice parameters to be *a*_m_ = 5.662 Å, *b*_m_ = 5.606 Å, *c*_m_ = 3.905 Å, and monoclinic angle β = 89.5°. The *b*_m_ axis is partially relaxed, and the *c*_m_ axis is fully strained (Fig. 2, B and C), as previously observed (*26*).

(100)_m_-oriented BiFeO_3_ films can show two different ferroelastic domain states, either with *c*_m_ parallel or perpendicular to the [001]_c_ axis of SrTiO_3_ (*26*). For each ferroelastic domain state, two opposite polarization directions are possible, giving rise to four polar domain states in total. XRD reveals that the film is ∼99.5% a pure single ferroelastic domain (see Methods and note S1). In addition, the polarization-dependent optical birefringence shows a sinusoidal shape with transmittance maxima for light polarizations parallel to *b*_m_ (Fig. 2D). As the SrTiO_3_ substrate is cubic and hence optically isotropic, the sinusoidal shape shows the macroscopic optical anisotropy of our BiFeO_3_ thin film and confirms the ferroelastic single-domain state.

To investigate the polar domain states within the ferroelastic domain, we used piezoresponse force microscopy (PFM) (Fig. 2, E to H; Methods; and note S1). The topography image exhibits uniaxial features parallel to the *c* axis of the substrate (Fig. 2E). The out-of-plane and in-plane PFM phase images are homogeneous (Fig. 2, F and G), which indicate a polar single-domain state. The out-of-plane component of the polarization points downward, and the in-plane component is antiparallel to [001]_c_ SrTiO_3_. We rotated the sample by 90° (Fig. 2H) and repeated the scan. The absence of an in-plane PFM signal confirms the orientation of the polar single-domain state. This ferroelectric polarization is switchable with a dc voltage of 7 V applied between the SrRuO_3_ electrode and the PFM tip (insets of Fig. 2, F and G).

### Time-domain Brillouin light scattering

Next, we explore the impact of an ultrashort light pulse on our single-domain BiFeO_3_ thin film. In our experiment, the light pulse impinges at normal incidence with a pump photon energy of 3 eV, which is larger than the BiFeO_3_ bandgap (see Methods) (*31*). The absorption of a light pulse leads inevitably to the excitation of a strain pulse (*32*, *33*) that propagates through the film perpendicular to its surface (*11*). The crystal symmetry of the BiFeO_3_ film (point-group *m*) allows for the generation of both longitudinal and transverse acoustic waves, i.e., out-of-plane and in-plane atomic motions, respectively.

**Fig. 3.**
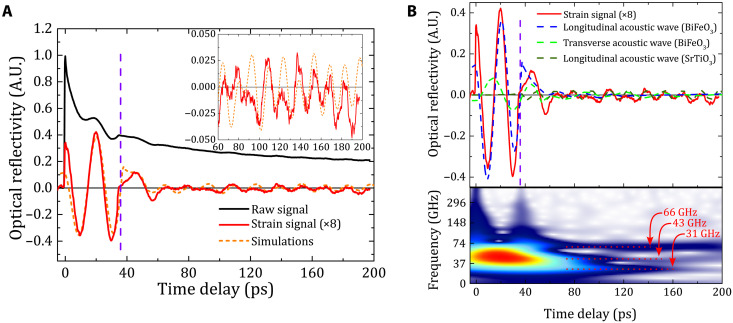
Time-resolved optical reflectivity. (**A**) Raw signal of the transient optical reflectivity (black curve) measured by time-resolved optical pump-probe experiments. The oscillatory coherent acoustic phonons signal is extracted from the raw signal (red curve). The purple line shows abrupt change in the oscillatory signal. The simulated oscillatory signal is shown by the orange dashed line. Inset: Zoom into the optical reflectivity for longer time delays. (**B**) Top: Comparison between the experimental acoustic signal (in red) and the simulated three acoustic modes (longitudinal, LA and shear, TA, acoustic modes in BiFeO_3_ and one longitudinal acoustic mode in SrTiO_3_, dashed lines) constituting the simulated acoustic signal in (A). Bottom: Wavelet analysis of the experimental acoustic signal. The colored map represents the experimental signal in the time-frequency domain and shows the three distinct acoustics modes at 31 GHz (TA mode in BiFeO_3_), 43 GHz (LA mode in BiFeO_3_), and at 66 GHz (LA mode in SrTiO_3_). The vertical purple dashed line in (A) and (B) indicates the time delay when the LA wave in BiFeO_3_ arrives at the BiFeO_3_-substrate interface.

In a first step, we probe the change of the optical reflectivity in a pump-probe measurements with picosecond resolution. This allows us to reveal the different photo-induced acoustic modes through time-domain Brillouin light scattering (*11*, *18*, *19*, *34*) (see Methods). The black curve in Fig. 3A shows the raw signal of the transient optical reflectivity as a function of time delay between the pump and the probe pulse. We subtract the electronic and thermal contributions of the raw signal and extracted the Brillouin oscillatory components of the time-domain optical reflectivity (Fig. 3A in red). To identify the oscillatory components, we conducted a standard Wavelet analysis on the oscillatory signal. This yields the signal strength in the frequency domain as a function of the time delay between the pump and the probe pulses (Fig. 3B, bottom). The data reveal three distinct modes at about 31, 43, and 66 GHz. To pinpoint the origins of these modes, we compare the mode frequencies with literature data on bulk BiFeO_3_ and SrTiO_3_ (see also note S2). We find that the mode frequencies match with the transverse (shear strain) and longitudinal Brillouin modes of bulk BiFeO_3_ and with the longitudinal Brillouin mode of SrTiO_3_, respectively. The deduced longitudinal (*v*_L_ ≈ 4500 m/s) and shear (*v*_T_ ≈ 3100 m/s) sound velocities in the BiFeO_3_ thin film are also consistent with the values found in bulk BiFeO_3_ (*17*, *18*). Next, we investigate the signal evolution in time. Until ~40 ps, the experimental optical signal is dominated by the longitudinal acoustic phonon signal of the BiFeO_3_ layer. At ~40 ps, the signal exhibits a discontinuity (dashed purple line in Fig. 3, A and B). This abrupt signal change corresponds to the arrival of coherent longitudinal acoustic phonons at the BiFeO_3_-substrate interface (*35*–*38*). Thanks to a small acoustic reflection coefficient (see note S3), this acoustic mode is largely transferred into the substrate. After this acoustic transmission, the transverse acoustic mode dominates the experimental Brillouin oscillatory signal in the BiFeO_3_ layer until roughly 60 ps. At this time, the transverse mode is partly transferred into the substrate and partly reflected at the BiFeO_3_-substrate interface. Note that due to our measurement configuration, we can only probe the transmitted longitudinal acoustic wave in the cubic SrTiO_3_ substrate (*39*).

To gain further insights, we have simulated the experimental strain signal in two steps. First, we model the lattice dynamics of the BiFeO_3_ thin film after the short laser pulse excitation by semicoupled equations of motion. This allows the evaluation of the dynamics of coherent acoustic phonons in the structure (see note S3) (*40*). Second, the transient optical reflectivity is calculated on the basis of the standard photoelastic model (see Methods and note S3). In this simulation, because we do not know the photoelastic coefficients (for the longitudinal and the shear waves) involved in the Brillouin light scattering detection process in BiFeO_3_ and SrTiO_3_, we used them as free parameters to adjust the amplitude of the longitudinal and shear waves. The simulation results are displayed as dashed orange lines in Fig. 3A. The simulations are in excellent agreement with the experimental results even at longer time scales (inset of Fig. 3A). In Fig. 3B (top), we displayed the different waves that compose the total simulated signal as dashed lines. Hence, we can identify the different time windows where one specific acoustic mode dominates the signal.

These preliminary all optical experiments demonstrate the possibility to light-induce longitudinal and transversal strain waves in our single ferroelectric domain BiFeO_3_ thin film. Next, we build up a detailed understanding of the evolution of the strain waves.

### Time-resolved XRD

**Fig. 4.**
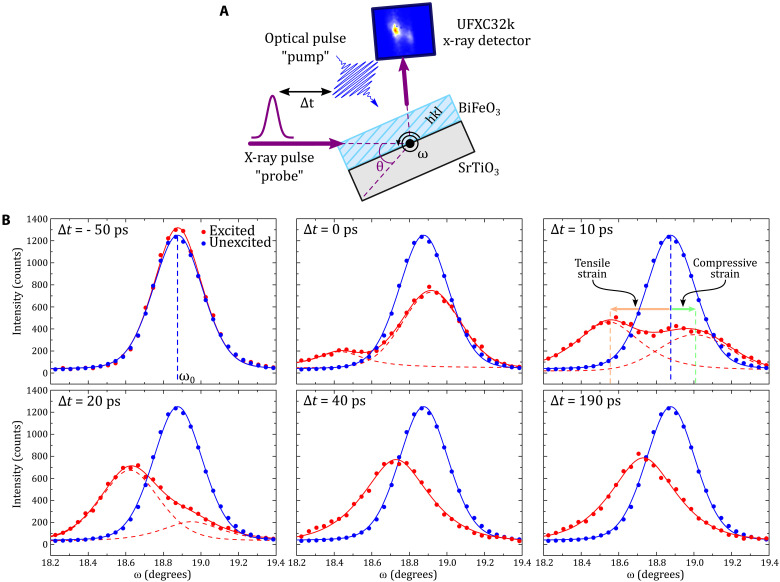
Time-resolved XRD experiments. (**A**) Sketch of the time-resolved XRD setup. Rocking curves (ω scans) were measured to record the evolution of the Bragg peak positions as a function of the time delay (Δ*t*) between the optical pump pulse and the x-ray probe pulse. The SrRuO_3_ buffer layer is omitted for clarity. (**B**) Transient evolution of the 40${\bar{3}}_{m}$ Bragg peak (red dots) after ultrashort laser pulse excitation compared to the reference unexcited Bragg peak (blue dots). The lines are the results of the fitting procedure (Methods).

The time-domain Brillouin light scattering can probe acoustic phonons through the photoelastic interaction but cannot provide directly the amplitude of the photo-induced strain (*41*). In the next step, we quantitatively evaluate the strain amplitude within the unit cells. To do so, we turn to time-resolved XRD. This technique allows us to monitor the photo-induced strain pulse through the evolution of the Bragg peak positions with time (*9*–*12*, *42*). First, we have to identify suitable Bragg reflections. Here, a look on symmetry properties is insightful. With the point-group symmetry (*m*) of BiFeO_3_, the mirror plane lies perpendicular to the *b*_m_ axis and contains the *a*_m_ and *c*_m_ axes. Hence, the photoexcitation cannot lead to in-plane atomic displacements along *b*_m_ due to symmetry principles (see note S4). In other words, the photo-induced stress must have two components (*11*, *18*, *33*, *43*): an out-of-plane and in-plane components. These two stress components cause then atoms to move simultaneously along out-of-plane and in-plane directions (*11*, *18*, *33*, *43*). Accordingly, photo-induced deformations comprise two strain components: the longitudinal strain η_L_, which describes the expansion or contraction along *a*_m_, and the shear strain η_T_ with atomic displacement along *c*_m_ (Fig. 1). Diffraction measurements with Bragg planes (*h*0*l*)_m_ and (${h0\bar{l})}_{m}$ type gives access simultaneously to the longitudinal and shear strain (*11*). In contrast, the (*hk*0)_m_/($h\bar{k}0$)_m_ planes are only sensitive to the longitudinal strain (see note S4). From an experimental point of view, we monitored the photo-induced transient evolution of different Bragg peak positions by so-called ω scans (i.e., rocking curves; see Fig. 4A, Methods, and note S4). In this geometry, the η_L_ and η_T_ strains translate into diffraction angles ω as follows

$$\begin{array}{rl} {\left(\omega^{\prime }-\omega_{0}\right)}_{h0l} & =-A(\theta ,h,|l|)\left[\eta_{L}\frac{h}{|l|}+\sqrt{2}\eta_{T}\right]\\ {\left(\omega^{\prime }-\omega_{0}\right)}_{h0\bar{l}} & =-A(\theta ,h,|l|)\left[\eta_{L}\frac{h}{|l|}-\sqrt{2}\eta_{T}\right]\\ {\left(\omega^{\prime }-\omega_{0}\right)}_{hk0} & ={\left(\omega^{\prime }-\omega_{0}\right)}_{h\bar{k}0}=-B(\theta ,h,|k|)\eta_{L} \end{array}$$ (1)

where ω′ and ω_0_ denote the time-dependent diffraction angle and the diffraction angle at equilibrium, respectively. *A*(θ, *h*, ∣l∣) and *B*(θ, *h*, ∣k∣) are positive crystallographic parameters. Details about the derivation of Eq. 1 can be found in the note S4. As we see in Eq. 1, measuring ω′ − ω_0_, with these set of Bragg peaks, allows us to reconstruct fully the light-induced strain evolution of η_L_(*t*) and η_T_(*t*) at the picosecond time scale.

Equipped with these preliminary considerations, we embark to investigate the photo-induced strain in our BiFeO_3_ thin film. We select the Bragg reflections of the (40$\bar{3}){}_{m}$ and (403)_m_ planes to access the longitudinal and shear strain distortions. In addition, we measure the Bragg reflections of the (530)_m_ and (5$\bar{3}$0)_m_ planes as a reference for distortion of the longitudinal strain only. Figure 4B shows representative ω scans of the 40${\bar{3}}_{m}$ Bragg peak for selected time delays between ~50 and 190 ps (red curves). The blue curve is the reference Bragg reflection of the unperturbed system. The transient evolution of the 40${\bar{3}}_{m}$ Bragg peak profile reveals a complex behavior. First, within the first ~30 ps, the Bragg peak is highly asymmetric and is split into two contributions. Second, at longer time scale, only one Bragg peak exists and remains shifted to a lower angle up to the maximum measured time delay (~200 ps). This general trends is also shared by the 403_m_, 530_m_, and 5$\bar{3}$0_m_ Bragg reflections.

**Fig. 5.**
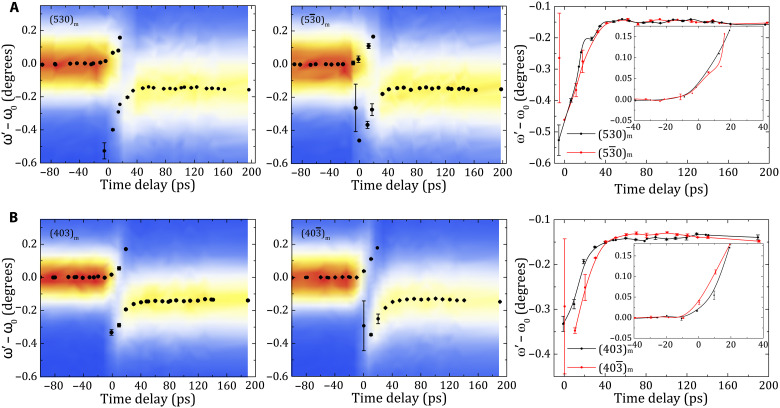
Time-resolved light-induced strain. (**A** and **B**) Maps of the Bragg peak position change (ω′ − ω_0_) recorded as a function of the time delay for 530_m_ and 5$\bar{3}$0_m_ reflections (A) and 403_m_ and 40${\bar{3}}_{m}$ reflections (B). The right panels show the temporal evolution of the Bragg peaks positions change for the tensile and the compressive parts (inset) for the 530_m_/5$\bar{3}$0_m_ (top) and 403_m_/40${\bar{3}}_{m}$ reflections (bottom).

For an in-depth analysis of the evolution of Bragg reflections, we have extracted the diffraction angles by a standard fitting procedure (Methods). Depending on the time delay, we have two or one Bragg peak to fit. Figure 5 (A and B) shows the ω′ − ω_0_ evolution for the four reflections as a function of the time delay. At first glance, the diffraction patterns of all four planes show similar behaviors. Past the time delay zero, the Bragg peak splits into two distinct Bragg reflections (later called negatively and positively shifted Bragg peaks), as shown in Figs. 4B and 5 (A and B). These two Bragg peaks indicate that, in the early moments, the BiFeO_3_ layer consists of two distinct regions: one under compressive strain [(ω′ − ω_0_) > 0] and the other under tensile strain [(ω′ − ω_0_) < 0], as we will describe later. One Bragg reflection [(ω′ − ω_0_) > 0] shows a steady shift from ω_0_ to ω′ − ω_0_ ≈ 0.2° and disappears at ~30 ps. In contrast, the second Bragg reflection [(ω′ − ω_0_) < 0] appears with the time delay zero at ω′ − ω_0_ ≈ −0.4°. The difference in ω′ − ω_0_ decreases over the first ~30 ps to ω′ − ω_0_ ≈ −0.15°, where it stabilizes for the maximal time delay of our measurements of ~200 ps. A closer look confirms the nearly identical temporal evolutions of the (530)_m_ and (5$\bar{3}0$)_m_ Bragg reflections (Fig. 5A, right; note the apparent difference the (530)_m_ and (5$\bar{3}0$)_m_ reflection around time delay zero results from the sharp signal change). Yet, the 403_m_ and 40${\bar{3}}_{m}$ Bragg peak shifts exhibit distinct changes in their temporal evolutions (Fig. 5B). In the time range where two Bragg peaks are detected (0 to 30 ps), whatever the peak we consider [(ω′ − ω_0_) > 0 or (ω′ − ω_0_) < 0], the shift of the 40${\bar{3}}_{m}$ Bragg peak is always more pronounced (positively or negatively) than the shift of the 403_m_ Bragg peak. For time delays of 40 to 120 ps, where we detect only one Bragg peak, being negatively shifted regarding to ω_0_, this behavior is reversed and the 403_m_ Bragg peak shift becomes larger than that of the 40${\bar{3}}_{m}$. Last, the diffraction angles of the 403_m_ and 40${\bar{3}}_{m}$ planes converge toward 200 ps. Crucially, the different behavior observed between 40${\bar{3}}_{m}$ and 403_m_ in Fig. 5B (right) proves that shear strain contributes to the Bragg shift, consistently with the model presented in Eq. 1.

To extract the longitudinal and shear strain amplitudes quantitatively, we rewrite Eq. 1, expressing the longitudinal strain η_L_ and shear strain η_T_ in terms of a combination of the ultrafast light-induced changes of the diffraction patterns

$$\begin{array}{rl} \eta_{L} & =\frac{-|l|}{2hA(\theta ,h,|l|)}[{\left(\omega^{\prime }-\omega_{0}\right)}_{h0l}+{\left(\omega^{\prime }-\omega_{0}\right)}_{h0\bar{l}}]=\frac{-1}{B(\theta ,h,|k|)}{\left(\omega^{\prime }-\omega_{0}\right)}_{h\pm k0}\\ \eta_{T} & =\frac{-1}{2\sqrt{2}A(\theta ,h,|l|)}[{\left(\omega^{\prime }-\omega_{0}\right)}_{h0l}-{\left(\omega^{\prime }-\omega_{0}\right)}_{h0\bar{l}}] \end{array}$$ (2)

**Fig. 6.**
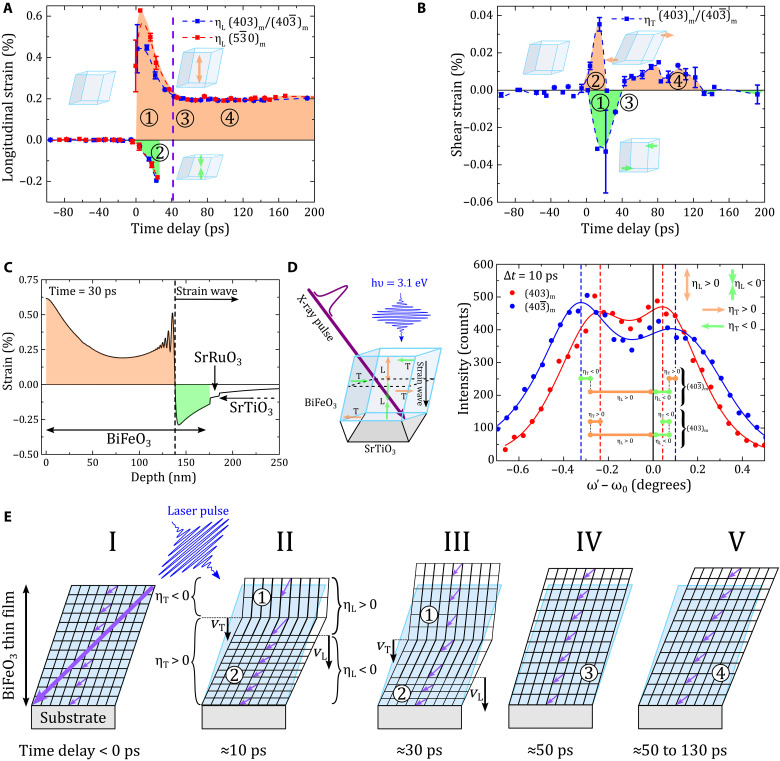
Light-induced longitudinal and shear strains. (**A** and **B**) Time evolution of the longitudinal [η_L_ (A)] and shear [η_T_ (B)] strains extracted from the 403_m_/40${\bar{3}}_{m}$ and 530_m_/5$\bar{3}0_{m}$ Bragg reflections. The color filling [orange and green in (A) and (B)] and the small sketches in (A) and (B) indicate the unit cell distortions. The purple dashed line shows the approximate arrival time of the out-of-plane strain pulse to the BiFeO_3_-substrate interface. (**C**) Simulation of the spatial dependence of the longitudinal strain in the sample at 30 ps after ultrashort laser pulse excitation. The profile shows the compressive front (green) and the tensile tail (orange) of the strain pulse propagating at the speed of sound. These opposite strain components lead to the two diffraction peaks as shown in (D) and Fig. 5B. (**D**) Left: Sketch of the light-induced strain components (longitudinal-L and shear-T) within the BiFeO_3_ film illustrating the compressive front and tensile tail of the L and T strain pulses. Right: The 403_m_ and $40{\bar{3}}_{m}$ Bragg peaks at 10-ps time delay. The different shifts of the two Bragg peaks are related to the L and T strain contributions. Note that the shear strain component has an opposite contribution for 403_m_ and $40{\bar{3}}_{m}$ consistently with Eq. 1. (**E**) Temporal and spatial dependence of the light-induced strain in BiFeO_3_ thin film and its effect on the ferroelectric polarization. The longitudinal and shear strains travel at velocities *v*_L_ and *v*_T_, respectively, distort the unit cell and the local unit-cell ferroelectric polarization direction (small purple arrows) as a function of the time delay. The uniform macroscopic ferroelectric polarization before laser excitation is shown by the large purple arrow (sketch I).

We use the experimental data (ω′ − ω_0_)_403_m__ and ${\left(\omega^{\prime }-\omega_{0}\right)}_{40{\bar{3}}_{m}}$ shown in Fig. 5B to calculate the longitudinal and shear strains, which are displayed in Fig. 6 (A and B, respectively). We find that tensile (label 1 in Fig. 6A) and compressive (label 2 in Fig. 6A) longitudinal strain components coexist for the first 10 to 30 ps, consistently with the coexistence of positively and negatively shifted Bragg peaks. We also note that the compressive and tensile longitudinal strains deduced from the hk0_m_ or from the combination of h0l_m_ and h0${\bar{l}}_{m}$ reflections have the same overall time dependence, which directly demonstrates that the extraction of the longitudinal strain does not depend on the selected Bragg peak, thus confirming the validity of the model presented in Eqs. 1 and 2. From the negatively shifted Bragg peak, we determine a maximal longitudinal strain of 0.6%. In the same time range, we also determine the shear strain amplitude, which we can extract from both the negatively (label 1 in Fig. 6B) and positively shifted (label 2 in Fig. 6B) Bragg peaks. We obtain 0.04% as the largest amplitude of the shear strain. The coexistence of positive and negative longitudinal and shear strains can be explained and simulated by the standard elastic response of a solid object to a photo-induced stress (see Fig. 6, C and D), which has been previously introduced to simulate the time-domain Brillouin light scattering experiment (Fig. 3 and note S3). When the light is absorbed in the bulk of the BiFeO_3_ layer near the surface, the light-induced stress generates a lattice strain pulse that propagates in both forward and backward directions (*32*). When the backward emitted strain pulse arrives at the mechanically free air-BiFeO_3_ interface, it is reflected and the sign of the strain reverses. As a result, the propagating strain pulse becomes bipolar (note S3). For example, if the initial longitudinal stress is negative (as in the case of thermal expansion), then the front of the strain pulse is compressive and its tail is tensile. Figure 6C illustrates this scenario with the simulation of the spatial dependence of the longitudinal bipolar strain pulse as it travels through the BiFeO_3_/SrRuO_3_/SrTiO_3_ system 30 ps after photoexcitation. In our experiment, the x-rays penetrate and probe the entire BiFeO_3_ layer at once, so that they can detect the regions exhibiting different strain fields simultaneously. This explains the coexistence of a compressive and tensile strain in the time-domain measurements shown in Figs. 6A-B, i.e., why we have a splitting of the Bragg peak in the early time-delays. Similar light-induced bipolar longitudinal strain waves have also been observed in other materials, such as InSb (*42*) and SrRuO_3_ (*44*). Fig. 6D illustrates the relationship between the negatively and positively shifted Bragg peaks and the positive and negative longitudinal strain present in the tail and front of the strain pulse, respectively. As previously mentioned, light also induces shear stress, which generates a bipolar shear strain pulse within the film (see note S3). This additional contribution of the shear strain components to the Bragg peak shift has a different sign in the front and the tail of the strain pulse and has a different impact on the 403_m_ and 40${\bar{3}}_{m}$ peaks as shown in Fig. 5B and summarized in Fig. 6D. This is in perfect agreement with our model presented in Eq. 1.

These results are worth emphasizing for two reasons. First, to the best of our knowledge, the longitudinal strain level achieved in this study is among the largest photo-induced longitudinal strain levels ever reported in BiFeO_3_ film. Similarly, high strain levels have been reported recently but only in multidomain BiFeO_3_ film (*15*). This efficient photo-induced strain is likely connected to efficient generation mechanisms of acoustic waves that combine light-induced thermal (ultrafast thermal expansion) and nonthermal (deformation potential mechanism, inverse piezoelectric effect) processes, as already discussed in the literature for the rhombohedral BiFeO_3_ (*9*–*11*, *15*, *18*). Second, it is the first observation and evaluation of ultrafast light-induced shear strain component in ferroelectric thin films. Previous works, for example, on BiFeO_3_ and PbTiO_3_ thin films, targeted the out-of-plane motion only (*9*, *10*, *12*, *14*, *15*, *20*).

In the next step, we can have a look at the details of the evolution of the longitudinal and shear strains as they propagate through the sample. Let us start with the longitudinal strain pulse (Fig. 6A). The compressive strain (label 2 for the front) increases as the strain pulse propagates toward the BiFeO_3_-substrate interface (see Fig. 6A and fig. S5) and reaches a maximum strain level at around 20 ps, where it vanishes. The tensile longitudinal strain region (label 1 for the tail) emerges as a steep increase and reaches its maximum amplitude within the first 10 ps. Subsequently, it decays and reaches a plateau at ~40 ps (denoted by the purple dashed line in Fig. 6A). Because of the small acoustic reflection coefficient at the interface, the longitudinal strain wave is transmitted almost entirely into the substrate after ~40 ps (*R*_L_ ≈ 2%; note S3). The drop of the longitudinal strain at ~40 ps is perfectly consistent with the drop in the Brillouin oscillatory signal of the longitudinal mode in BiFeO_3_ in Fig. 3. This efficient strain transmission also explains why the compressive component (front) disappears and why only one Bragg peak remains after ~40 ps. The constant plateau-like longitudinal strain state after ~40 ps (labels 3 and 4 in Fig. 6A) results then from an overall expansion of the thin film caused by long-living and nonpropagating (at this time scale) thermal and/or electronic contributions (*9*, *11*, *18*). Next, we investigate the evolution of the shear strain (Fig. 6B). We remind that the shear strain is extracted by combining the nonequivalent responses of the 403 _m_/40${\bar{3}}_{m}$ reflections (Figs. 5B and 6D) as explained in the second term of Eq. 2. The maximal amplitude of the shear strain is an order of magnitude smaller than that of the longitudinal strain. Directly after the time delay zero, the front of the shear strain pulse (label 2) shows a positive amplitude, i.e., the monoclinic distortion increases. This shear-pulse front vanishes at ~20 ps. On the contrary, the tail of the shear strain pulse (label 1) is negative from 0 to ~50 ps. Accordingly, the monoclinic distortion decreases. At ~50 ps, this propagating shear strain changes sign and the monoclinic distortion increases again. It remains positive up to the time delay of ~130 ps, where it vanishes (labels 3 and 4). The absence of the shear strain signal beyond 130 ps, i.e., the absence of a plateau-like behavior at long time scale, indicates a rather small contribution to the shear strain of the thermal and/or the long-living electronic processes. This positive shear strain from 50 to 130 ps may have two origins. (i) The reflection coefficient of the shear acoustic wave at the BiFeO_3_-substrate interface has a non-negligible negative value (*R*_T_ ≈ −10%; note S4). Such a negative reflection coefficient can lead to a reverse of the strain sign when the tail of the shear strain impinges on the substrate. (ii) As BiFeO_3_ is elastically anisotropic, a mode conversion of the incident longitudinal wave into a shear wave may also occur (*43*). With a longitudinal strain 10 times larger than the shear strain, even a few percent of efficiency of this mode conversion could give rise or contribute to the shear strain between 50 and 130 ps.

## DISCUSSION

Figure 6E provides a sketch of the temporal and spatial tracking of the longitudinal and shear strain fields. To help the reader, we label the sketches I to V in Fig. 6E. We link deformations of the film in Fig. 6E to the strain states in Fig 6 (A and B) with the labels 1 to 4. Sketch I represents the unperturbed monoclinic BiFeO_3_ film before the arrival of the light pulse. In sketch II, the light pulse has excited the strain pulse with longitudinal and shear components traveling through the BiFeO_3_ film at their respective velocities *v*_L_ and *v*_T_. In the tail (label 1), the combination of a positive longitudinal and negative shear strains leads to an expansion of the unit cell along *a*_m_ together with a decrease of its monoclinicity. In the front (label 2), the longitudinal and shear strain have opposite signs. This leads then to a contraction of the unit cell along *a*_m_ together with an increase of its monoclinicity. As time goes on (sketch III), the strain pulse travels further through the BiFeO_3_ film and the longitudinal and shear components arrive at the interface at around 40 and 50 ps, respectively. In sketch IV, the longitudinal strain pulse has been transferred to the substrate. Only the long-living longitudinal strain component remains. Hence, the unit cell remains expanded along *a*_m_ (label 3). At this moment (~50 ps, label 3), the shear strain is nearly zero. Past this moment, the shear strain experiences a sign change and remains positive up to ~130 ps along with the long-living expansion along *a*_m_ (label 4 and sketch V).

In BiFeO_3_ films, the electric polarization is locked to the lattice, which is a fundamental property for ferroelectrics. This so-called adiabatic approximation is relevant even at the picosecond time scale as supported by theoretical calculations (*45*) and also recently discussed in ferroelectric superlattices submitted to a picosecond strain pulse (*28*). Assuming that the ferroelectric polarization equally follows the strain wave, as sketched in Fig. 6E (purple arrows), this entails an electric charge wave propagating at the speed of sound through the BiFeO_3_ film. The charge wave may even include electrostatic discontinuities because of the bipolar nature of the propagating strain wave. Considering the fast dynamics and the fundamental relation between a transient polarization and the current ($\vec{J}=\frac{\partial \vec{P}}{\partial t}$), we can expect ultrafast strain-induced current approaching the terahertz range. This is a promising property for next-generation ferroelectric-based gigahertz-terahertz technologies. The full description of this charged propagating acoustic front requires additional experiments and is planned future work.

In summary, we have used a combination of time-resolved XRD and optical ultrafast spectroscopy experiments to unravel the optically induced picosecond strain dynamics of a single-domain BiFeO_3_ thin film. We report maximum longitudinal and shear strain of 0.6 and 0.04%, respectively. These strain levels are comparable to those of commercially available electrically driven transducers. However, the important point is that the strain pulse here has a typical duration of tenths of picoseconds, corresponding to a typical bandwidth of 100 GHz, while electrically driven piezotransducers typically operate in the megahertz regime. This demonstrates that efficient conversion of light energy into mechanical energy is also possible at the picosecond time scale. We have also demonstrated the complex temporal and spatial profile of both the out-of-plane and in-plane strain components. As a result, the ultrafast light-induced strain dynamics modulate the ferroelectric polarization direction in time and space within the film. This work constitutes an important milestone toward the exploration of the rich lattice dynamics in complex ferroelectric textures, ranging from mosaic to self-organized periodic structures (*46*–*50*). Moreover, ultrafast light-induced anisotropic strains might be used to create new transient phases or to generate ultrashort current pulses based on strain-induced modulation of the polarization direction. In the quest for the ultrafast manipulation of the polar and magnetic orders in multiferroic compounds such as BiFeO_3_, future work will require considering the dynamics of the unit-cell strain to account for comprehensive (inverse) piezoelectric and/or (inverse) magnetostrictive effects at the picosecond time scale.

## METHODS

### Epitaxial thin-film growth

A single-domain epitaxial BiFeO_3_ thin film was grown by pulsed laser deposition on a SrRuO_3_-coated (110)_pc_ SrTiO_3_ substrate using conditions reported previously (*29*, *30*). Ablation was carried out by a KrF laser with wavelength λ = 248 nm at 5 Hz. The 12-nm layer of SrRuO_3_ was grown from a stoichiometric target at 660°C in 100 mtorr of oxygen with fluence of ∼2 J/cm^2^. The BiFeO_3_ layer was grown from a Bi_1.1_FeO_3_ ceramic target, while the substrate was held at 590°C in a background oxygen pressure of 100 mtorr. The thickness of the BiFeO_3_ layer is estimated at 180 ± 10 nm, extrapolated from the growth rate, taken from a sample grown directly beforehand on which x-ray reflectivity calibration was performed.

### XRD structural characterization

The film was characterized using XRD using an Xpert Pro Materials Research Diffractometer (MRD) using Cu K_α1_ radiation from a two bounce Ge (220) monochromator (λ = 1.6504 Å) and one-dimensional (1D) detector (PIXcel). Single-phase growth was confirmed by standard coupled θ-2θ scans, while the domain structure and lattice parameters were determined by reciprocal space maps (RSMs) near the 220_c_, 221_c_, and 310_c_ SrTiO_3_ reflections (fig. S1). In the (110)_c_ SrTiO_3_ orientation, only two ferroelastic domains are possible (with four total ferroelectric directions) (*26*). The relative domain population of ferroelastic domains was determined by fitting 2D Gaussians to the BiFeO_3_ peaks in the RSM around the 221_c_ SrTiO_3_ reflection (fig. S2). The film was thus determined to have a 99.5%/0.5% volume fraction, making, it in a practical sense, a single domain.

### Piezoresponse force microscopy

PFM experiments were performed in a Nanoscope V multimode (Bruker) using an external ac source (DS360, Stanford Research) to excite the BiFeO_3_ thin film at a frequency of 35 kHz (far off resonance) with typical ac voltage excitations of 2 V peak to peak and external lock-in amplifiers (SR830, Standford Research). We used Cr/Pt-coated tips with a cantilever stiffness of 40 N/m. Two different orientations of the cantilever were used for the in-plane PFM measurements (Fig. 2, G and H) to characterize the in-plane domain structure of the films (fig. S3). The single ferroelectric domain could be switched with +7 V applied to the SrRuO_3_ electrode while scanning with the slow scan axis along [001]_c_, resulting in another single domain within the same ferroelastic domain (switching by 180°) (Fig. 2).

### Optical birefringence measurement

The optical birefringence was measured in transmission geometry with a 400-nm laser [photon energy of 3.1 eV, above the bandgap of BiFeO_3_ to ensure absorption (*31*, *51*–*53*)] with an unfocused beam with a diameter of ~2 mm.

### Time-domain Brillouin light scattering

Time-domain Brillouin light scattering was conducted at IMMM with a Ti:sapphire femtosecond laser oscillator (*11*, *17*–*19*, *34*). The oscillator delivered a 120-fs beam at 830 nm, which was split into two beams, the first (pump) being doubled in frequency (λ_pump_ = 415 nm) in a BaB_2_O_4_ crystal. The second beam (probe) is used to synchronously pump an optical parametric oscillator delivering a femtosecond laser beam in the visible range (λ_probe_ = 587 nm) below the bandgap of BiFeO_3_, which permits the probe beam to penetrate in the film and in the substrate and hence to detect the propagating strain pulse. The transient optical reflectivity was measured through a balanced photodiode scheme. Under this condition, the optical detection process reveals only Brillouin components of the light-induced strain field (*17*–*19*, *34*). These Brillouin oscillation frequencies are given by $f_{B}=2\;{nv}_{s}\lambda_{\mathrm{probe}}^{-1}$ (for a probe beam with a normal incidence), where *n* is the refractive index of the probed medium and *v*_s_ is the sound velocity (different for longitudinal or transverse acoustic modes).

### Time-resolved XRD

The time-resolved XRD experiment was performed at the CRISTAL beamline of SOLEIL synchrotron. Optical excitation was achieved with 400-nm laser pulses of <80-fs duration as obtained by frequency doubling the fundamental output of the laser system (Micra oscillator and Legend Elite Duo HP amplifier, Coherent). The photo-induced lattice deformations were probed using x-rays of 7.082-keV energy, selected by means of a Si(111) double-crystal monochromator. The overall time-resolution of the experiment is set by the x-ray pulse duration, which is 10-ps full width at half maximum, as the storage ring was operated in the low-α mode (*54*). The pump beam irradiates nearly entirely the sample surface, while the probe x-ray beam is made at least twice smaller (maximum size of the x-ray footprint on sample: 90 μm by 500 μm). The various Bragg reflections studied were imaged using a photon-counting camera that was newly developed at SOLEIL, which enables simultaneous measurements at positive and negative pump-probe delays (*55*). Bragg reflections with high Miller indices (such as 403_m_ and 530_m_) were chosen to increase the sensitivity of the measurement to the light-induced strain. The rocking curves were fitted with either one or two pseudo-Voigt functions.

### Light-induced strain calculations

Light-induced strain calculations were done using the udkm1Dsim toolbox in Python, which simulates the thermal and the structural dynamics after light excitation of a 1D layered structure (*40*). The complete thermal and structural dynamics of the BiFeO_3_/SrRuO_3_/SrTiO_3_ nanostructure were modeled, although the strain of SrRuO_3_ layer is not displayed in the manuscript, especially in Fig. 3, as it is rather small. Further details can be found in note S3.

## Supplementary Materials

### This PDF file includes:

Figs. S1 to S9 Tables S1 and S2 Notes S1 to S5 References
