# Supplementary material for: Temporal and spatial tracking of ultrafast light-induced strain and polarization modulation in a ferroelectric thin film
Source: Sci Adv. 2023 Nov 15;9(46):eadi1160. doi: 10.1126/sciadv.adi1160 (PMC10651133; doi:10.1126/sciadv.adi1160)
Supplement: Supplementary file 1 — Figs. S1 to S9 Tables S1 and S2 Notes S1 to S5 References [file sciadv.adi1160_sm.pdf]

Science Advances
AAAS

Supplementary Materials for

Temporal and spatial tracking of ultrafast light-induced strain and polarization modulation in a ferroelectric thin-film

Ruizhe Gu *et al.*

Corresponding author: Vincent Juvé, vincent.juve@univ-lemans.fr; Daniel Sando, daniel.sando@canterbury.ac.nz; Vincent Garcia, vincent.garcia@cnrs-thales.fr; pascal.ruello@univ-lemans.fr

Sci. Adv. **9**, eadi1160 (2023)
DOI: 10.1126/sciadv.adi1160

This PDF file includes:

Figs. S1 to S9
Tables S1 and S2
Notes S1 to S5
References

I. SUPPLEMENTARY NOTE 1. SINGLE-DOMAIN CHARACTERIZATION

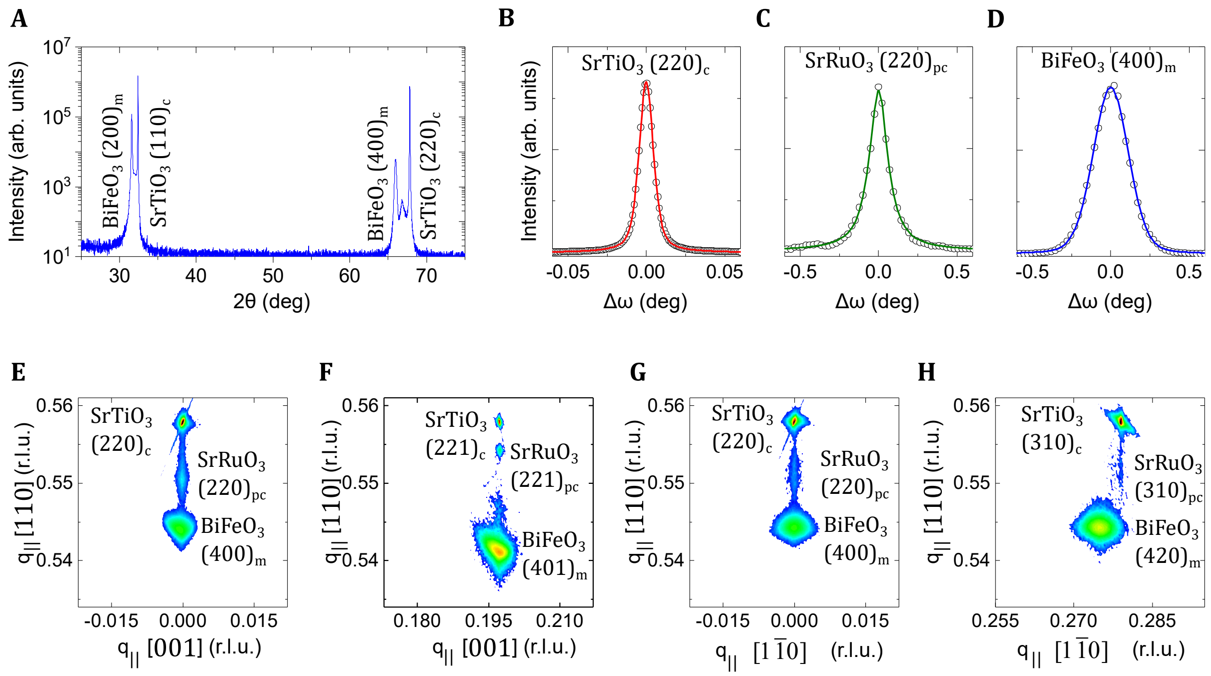

FIG. S1. **X-ray diffraction characterization of the single-domain $BiFeO_3$ thin film on $(110)_c$-oriented $SrTiO_3$. A,** θ-2θ scan. **B-D**, Rocking curves along the 220_c $SrTiO_3$ **(B)**, 220_{pc} $SrRuO_3$ **(C)** and 400_m $BiFeO_3$ reflections with full width at half maximum (FWHM) of 0.011°, 0.14°, and 0.266°, respectively. **(D). E-F,** Reciprocal space maps with in-plane momentum transfer along the $[001]_c$ direction of $SrTiO_3$ near the 220_c **(E)** and 221_c $SrTiO_3$ reflections **(F). G-H,** Reciprocal space maps with in-plane momentum transfer along the $[1\bar{1}0]_c$ direction of $SrTiO_3$ near the 220_c **(G)** and 310_c $SrTiO_3$ reflections **(H).**

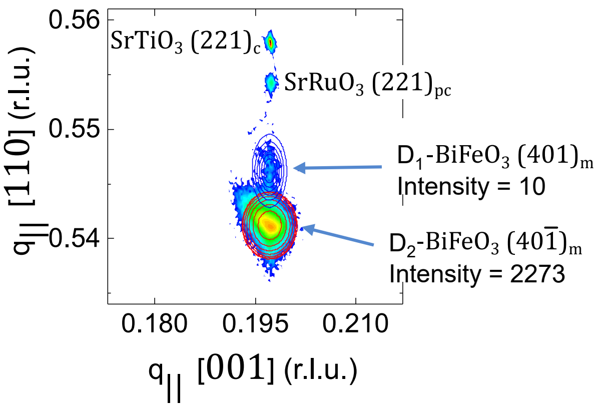

FIG. S2. **Estimation of the fraction of ferroelastic variants in the $BiFeO_3$ thin film on $(110)_c$-oriented $SrTiO_3$.** The integrated intensity of the two ferroelastic domains (D1 and D2) is calculated from the $(221)_c$ $SrTiO_3$ reciprocal space map.

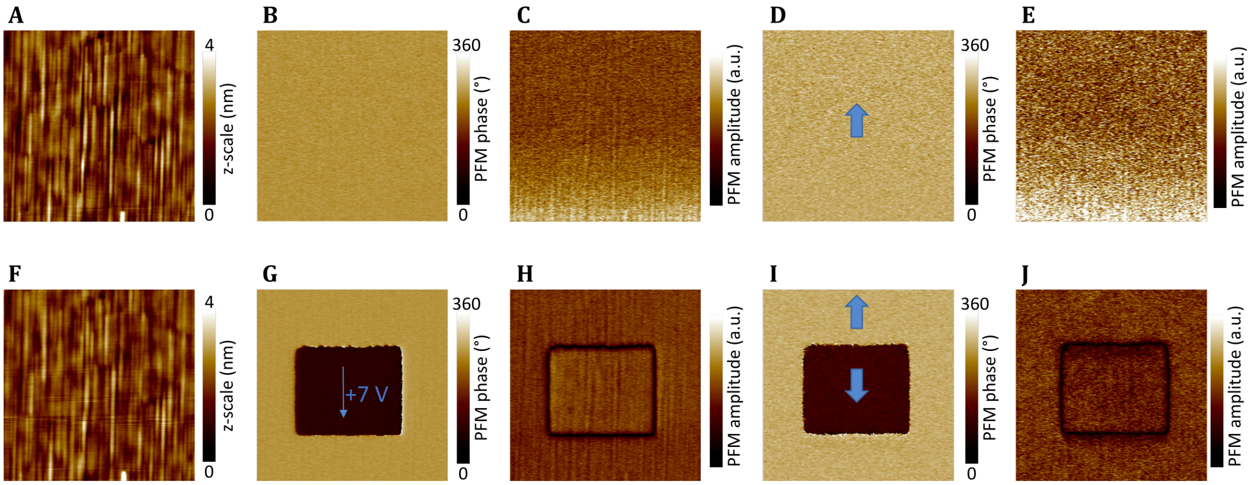

FIG. S3. **PFM characterization of the single-domain $BiFeO_3$ thin film on $(110)_c$ $SrTiO_3$.** **A-E,** As-grown state: topography **(A)**, out-plane PFM phase **(B)** and amplitude **(C)**, in-plane PFM phase **(D)** and amplitude **(E)**. **A-E,** Written state: topography **(F)**, out-plane PFM phase **(G)** and amplitude **(H)**, in-plane PFM phase **(I)** and amplitude **(J)**.

II. SUPPLEMENTARY NOTE 2. BRILLOUIN SIGNAL ANALYSIS

In this note we discuss in more details the values of the Brillouin frequencies, which we deduced from our Wavelet analysis shown in Fig. 3B of the main manuscript, and the corresponding sound velocities. In Fig. S4 we show typical acoustic phonon spectra extracted at different time delays (100, 115 and 175 ps). As mentioned in the main text, the Brillouin frequency of the LA phonon mode in $BiFeO_3$ is $f_{\mathrm{LA,\ BiFeO_3}} \approx 43\,\mathrm{GHz}$, in agreement with reported values in bulk single crystals and ceramic samples of rhombohedral $BiFeO_3$ (17, 18, 34). With a refractive index $n_{\mathrm{BiFeO_3}} \approx 2.9$ at $\lambda_{probe} = 587$ nm (51-53), the Brillouin frequency $f_{\mathrm{LA,\ BiFeO_3}}$ corresponds to a LA sound velocity of $v_{\mathrm{L,\ BiFeO_3}} = f_{\mathrm{LA,\ BiFeO_3}} \lambda_{probe} \cdot (2n_{\mathrm{BiFeO_3}})^{-1} \approx 4500$ m.s^{-1} in agreement with literature (15, 17, 56-58). The shear wave component in $BiFeO_3$ is observed at the Brillouin frequency $f_{\mathrm{TA}} \approx 31$ GHz, which leads to a sound velocity $v_{\mathrm{T,\ BiFeO_3}} = f_{\mathrm{TA,\ BiFeO_3}} \lambda_{probe} \cdot (2n_{\mathrm{BiFeO_3}})^{-1} \approx 3100\,\mathrm{m.s^{-1}}$ in good agreement with previous measurements on rhombohedral $BiFeO_3$ (17, 18, 57, 58). Finally, the LA wave in $SrTiO_3$ is detected with a Brillouin frequency of $f_{\mathrm{SrTiO_3}} \approx 66$ GHz. With the $SrTiO_3$ refractive index of $n_{\mathrm{SrTiO_3}} = 2.4$, we obtain the sound velocity $v_{L,\mathrm{SrTiO_3}} = 8100$ m.s^{-1}, in agreement with literature (59). In our work all sound velocities are given for a propagation direction of $(100)_m$. This corresponds in the pseudo-cubic to the $(110)_{pc}$ direction (see Figs. 1 and S6), so equivalent to the direction defined by the vector $(\vec{a} - \vec{b})$ where $\vec{a}$ and $\vec{b}$ are the two first unit cell vectors in the rhombohedral frame. Recently, along this direction (58) a longitudinal (LA) and shear (fast TA mode is the one we detect) velocities of $V_{LA} = 5100$ m.s^{-1} and $V_{TA} = 2700$ m.s^{-1} (see angle ϕ=0° in Figure 2 of Ref. (58)) have been measured. These values compare with our experimentally determined values given above ($V_{LA} = 4500$ m.s^{-1}, $V_{TA} = 3100$ m.s^{-1}).

There is around 13% of variation. This variation is of the same order than 5

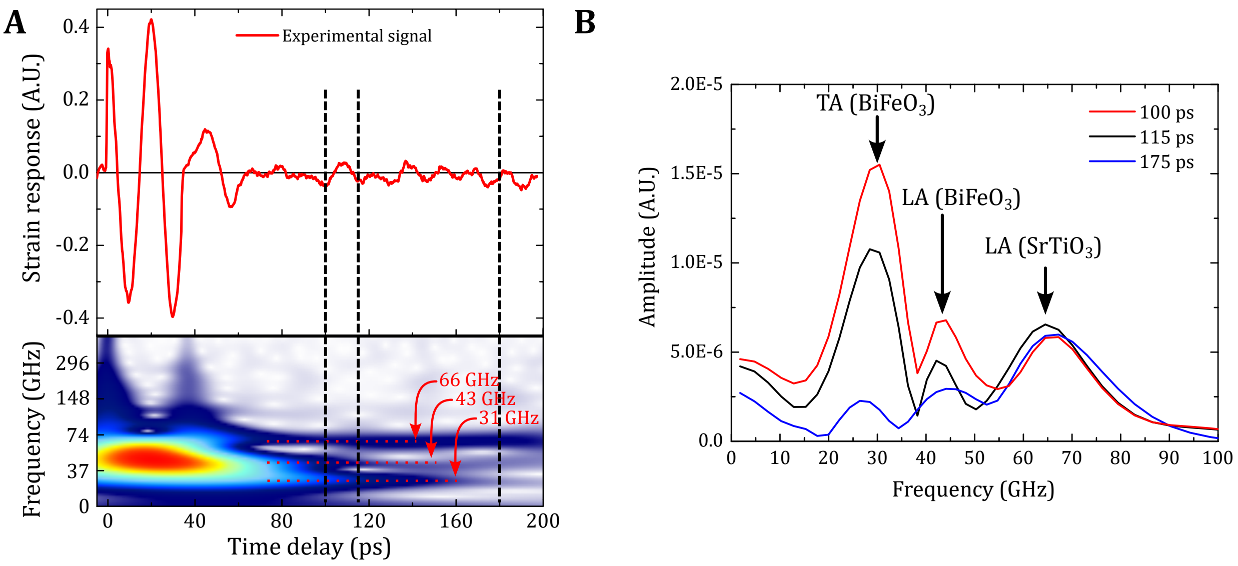

FIG. S4. **Acoustic phonon spectrum at different time-delays. A** Time-frequency domain map given by wavelet analysis as shown in Fig. 3B of the main manuscript. The vertical black dashed line indicates cuts in time (see **B**). **B** Spectral weight of the different acoustic phonon signals shown for different fixed time delays, displaying the three Brillouin modes.

those reported in the literature for bulk BFO where discrepancies between works approach sometimes 20 % (see Ref. (11, 18, 57, 58)).

III. SUPPLEMENTARY NOTE 3 : GENERATION, PROPAGATION, REFLECTION OF THE STRAIN WAVES IN THE $BIFEO_3/SRRUO_3/SRTIO_3$ FILM AND SIMULATION OF THE BRILLOUIN SIGNAL

A. Model of the photoinduced strain

When a material is subjected to a photoinduced stress, a strain pulse is generated in the material (semi-infinite geometry). Considering the simplest linear elastic response, we can assume that the photoinduced stress has the same form as the initial distribution of

light absorption with $\sigma = \sigma_0 e^{-\frac{z}{\xi}}$ where ξ stands for the light beam penetration depth. In the case of presence of light-induced longitudinal and shear stresses we can consider two kind of stress : $\sigma_L = \sigma_{0,z} e^{-\frac{z}{\xi}}$ and $\sigma_T = \sigma_{0,y} e^{-\frac{z}{\xi}}$. z stands as the direction of propagation which is perpendicular to the thin. In the case of $BiFeO_3$, the pump penetration depth is $\xi \sim 60$ nm for a pump wavelength of 400 nm and using the optical data known for the rhombohedral structure (31, 51-53). This penetration depth is smaller than the total layer thickness H=180 nm. The equation of motion, in the case of a longitudinal strain, is given by (32):

$$\frac{\partial^2 u_z}{\partial t^2} - v_L^2 \frac{\partial^2 u_z}{\partial z^2} = \frac{1}{\rho} \frac{\partial \left(\sigma_{0,z} e^{-\frac{z}{\xi}} \right)}{\partial z} \tag{S1}$$

where u_z(t,z), v_L, and ρ are the out-of-plane atomic displacement, the longitudinal sound velocity, and the mass density, respectively. In our experiment, it is indeed a one-dimensional problem since the lateral extension of the pump beam (spot size) is much larger (tens of micrometers) than the distance of propagation of acoustic waves in our study. For a time-delay of 200 ps, acoustic waves propagate typically over a distance of 1 micrometer only. As a consequence, the excitation has a piston-shape and the motion equation is 1D. Solving this equation for a semi-infinite system (with a mechanically-free surface at z=0 which corresponds to the $BiFeO_3$ surface) leads to the following general expression of the longitudinal strain ($\eta_L = \frac{\partial u_z}{\partial z}$) (32):

$$\eta_z(z,t) = \eta_0 \left(e^{-\frac{z}{\xi}} \left(1 - \frac{1}{2} e^{-\frac{v_L t}{\xi}} \right) - \frac{1}{2} e^{-\frac{|z - v_L t|}{\xi}} sgn\left(z - v_L t\right) \right) \tag{S2}$$

The formulation shown above for a longitudinal strain can be straightforwardly rewritten for a shear acoustic pulse with $\eta_T(z,t) = \frac{\partial u_y}{\partial z}$, and only the sound velocity has to be replaced by v_T. $u_y(z,t)$ is here the in-plane atomic motion propagating along z. In this model we assume, for simplification, that the longitudinal (LA) and shear (TA) waves are 7

pure LA and TA waves and we do not consider the possible quasi-longitudinal (QLA) and quasi-transverse (QTA) nature of these waves (11). Since the motion equation (Eq. S1) is a linear partial differential equation, the general solution can be a linear combination of a longitudinal and shear strains.

Once the strain pulse has left the region near the photoexcited surface, it propagates within the film and arrives to the interface $BiFeO_3$-$SrTiO_3$ where the propagating acoustic strain pulses undergo some reflection and transmission. To take into account these multiple reflections, we have simulated the evolution of a longitudinal and shear strains with the software *udkm1Dsim* developed by Schick *et al.*, available for free in the literature (40). In this calculation, the strain is modeled following the equation presented above (Eq. S1) with all the necessary optical parameters of the pump beam. The elastic properties of $BiFeO_3$, $SrTiO_3$ and those of the buffer layer $SrRuO_3$ are taken into account. The thermal diffusion in the $BiFeO_3$ layer, which can lead to an in-depth extension of the induced stress, is also taken into account but has negligible effect as already discussed previously so that the emitted strain pulse is very well described by Eq. S2 (10, 18). The calculation has been performed with the following acoustic velocities, as deduced from our Brillouin analysis (see Supplementary Note II): $v_{L,BiFeO_3}$=4.5 nm/ps, $v_{T,BiFeO_3}$=3.1 nm/ps, $v_{L,SrTiO_3}$=8.1 nm/ps and $v_{L,SrRuO_3}$= 6.3 nm/ps. The densities calculated by the program are ρ_{BiFeO_3}=8.8.10^3 kg/m^3, ρ_{SrRuO_3}=6.5.10^3 kg/m^3, and ρ_{SrTiO_3}= 5.2.10^3 kg/m^3. With this model, we can describe the propagation and reflection of both the longitudinal (LA) and shear (TA) strain pulses as a function of time. For the case of a longitudinal strain, the results are shown for different time delays in Fig. S5. In this calculation we can observe that there is a nearly perfect acoustic matching between the acoustic impedance of the LA waves and that of the substrate (see 8

also subsection B of Supplementary Note III for the discussion of the acoustic reflection coefficients). As a consequence, once the LA acoustic wave has travelled across the film, it is transmitted into the substrate and only the thermal component of the LA strain remains. Said differently there is no acoustic reflection of the LA waves on the substrate. For this reason, the compressive front (green area depicted in Figs. 6A and 6C) can be detected and analysed only during around 30 ps. We can see in Fig. S5 that the propagating front incident on the substrate and seen for a time delay of 40 ps, does not give rise to a reflected front at the time 40 ps since the elastic energy is indeed transferred into the substrate. By contrast, our calculations show that the shear (TA) waves do exhibit an acoustic mismatch with the substrate. As a consequence, these waves are reflected and are then confined in the film. Moreover, since the acoustic reflection coefficient for the TA waves is negative, there is a change of the sign of the strain perfectly in agreement with our observations (see Fig. 6B). The simulation of the strain field will be used in the part C of Supplementary Note III for the simulation of the transient optical reflectivity signal discussed in Fig. 3 in the main text. In this simulation we have taken into account the propagation of the longitudinal and shear acoustic phonons in $BiFeO_3$ and their reflections at the $BiFeO_3$-$SrTiO_3$ interface. We also simulate the transmitted longitudinal strain in $SrTiO_3$. As mentioned in the main text, we cannot detect for symmetry reasons the shear acoustic phonon in $SrTiO_3$ (39). As a consequence, we did not include it in the calculation. Finally we did not include the possible mode conversion (L to T and T to L) that could arise at the interface between an anisotropic ($BiFeO_3$) and isotropic ($SrTiO_3$) material when the incident longitudinal wave in $BiFeO_3$ impinges on the interface. Even if we cannot exclude a possible contribution of this mode-conversion, at this stage of the analysis it does not appear necessary to have a good agreement between the experimental time-resolved Brillouin signal and the simulated

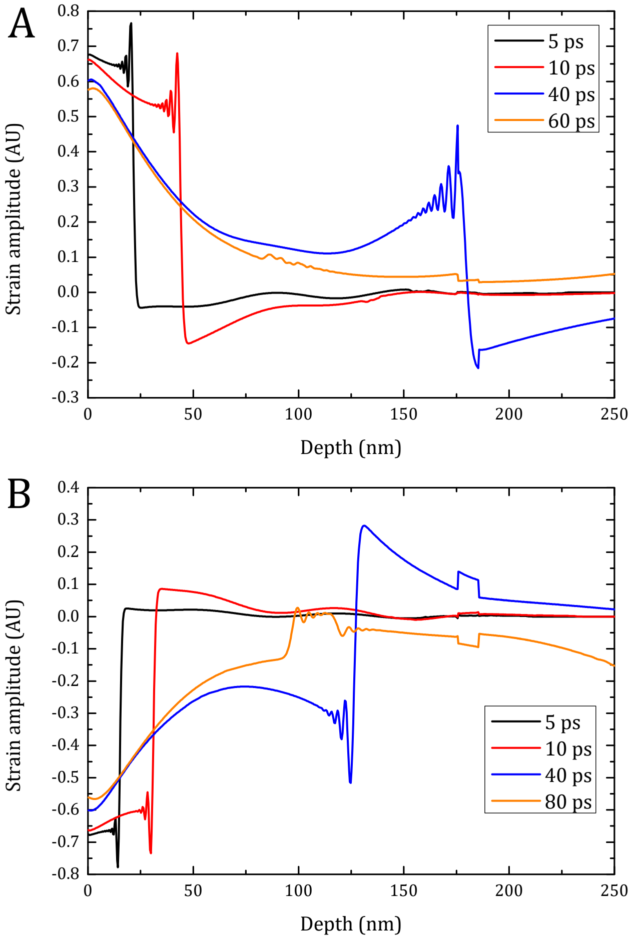

FIG. S5. **Numerical simulation of the time dependence of the light-induced strain in a $BiFeO_3$ thin film (180 nm).** Time-dependence of the longitudinal (**A**) and shear (**B**) strain in $BiFeO_3/SrRuO_3/SrTiO_3$ structure. Note that the longitudinal strain pulse (**A**) arrives at the interface between the film and the SRO-coated substrate at around 40 ps and is nearly entirely transmitted. For time-delay longer than 40 ps, only the long-living (non propagating) contribution remains (see Eq. S2). On the contrary, for the shear strain pulse (**B**), a non-negligible part is reflected at around 50ps. The reflected part of the shear strain pulse is visible at a time delay of $\sim$ 80 ps and is located at a depth of around 100-120 nm.

one as shown in Fig. 3.

B. Acoustic reflection

The acoustic reflection coefficient is given by

$$R = \frac{Z_2 - Z_1}{Z_2 + Z_1} \tag{S3}$$

where Z denotes the acoustic impedance with Z=$\rho \cdot v$, where ρ and v are the mass density and the sound velocity (either LA or TA), respectively. The subscript 1 defines the medium where the wave originates, while the subscript 2 denotes the medium from which the wave is reflected.

Considering the sound velocities determined from our experiment (Brillouin frequencies, see Supplementary Note II) and those from the literature for $SrRuO_3$, all summarized in Table S1, we have then estimated the respective acoustic reflection coefficients for the L and T waves. These values are shown in Table S2.

	$BiFeO_3$	$SrRuO_3$	$SrTiO_3$
ρ (m.s^{-1})	8220	6490	4810
v_L (m.s^{-1})	4500	6312 (60)	8200
v_T (m.s^{-1})	3200	3083 (60)	4580-4920 (61)
Z_L (P.s.m^{-1})	3.7.10^7	4.0.10^7	3.95.10^7
Z_T (P.s.m^{-1})	2.6.10^7	2.0.10^7	2.2-2.36.10^7

TABLE S1. Elastic parameters and density

Interface	R_L	R_T
$BiFeO_3/SrRuO_3$	0.03	-0.13
$BiFeO_3/SrTiO_3$	0.02	-0.07
Average	0.025	-0.10

TABLE S2. Acoustic reflection coefficients

As shown in Table S2, the reflection of the shear wave (R_T) at the first interface $BiFeO_3/SrRuO_3$ is notably larger than that of the longitudinal wave (R_L). The small R_L coefficient (around 2 %) is consistent with our observation of the absence of acoustic echo in the temporal evolution of the longitudinal strain presented in Fig. 6A (see main text). Our calculations show that R_T is negative independently on the nature of the interface ($SrRuO_3$, $SrTiO_3$), which is consistent with the change of sign of the shear strain signal in Fig. 6B.

In summary, these estimates predict a reversal of the shear strain upon reflection from the substrate/film interface, in agreement with our X-ray measurements (Figs. 6A and B) and in agreement with the simulation of the Brillouin signal discussed in Fig. 3. The simulation of the Billouin signal is detailed in the next subsection.

C. Simulation of the time-resolved Brillouin signal

The time and space dependence of the strain presented previously (Fig. S5) is used to simulate the transient optical reflectivity signal ($\Delta R/R$) shown in Fig. 3. In an optical pump-probe experiment, the strain modulates the refractive index (photoelastic effect or so-called Brillouin process) and also causes a motion of interfaces (interferometric effect). To simulate the contribution of the strain to our $\Delta R/R$ signal, we have applied the standard approach 12

already described in many previous works (35-38). In the particular case of the system studied in this work, since the buried $SrRuO_3$ layer is thin compared to both the $BiFeO_3$ layer and the probe wavelength (λ=587 nm), we assume that from the optical point of view the system can be considered as the assembly of a transparent layer ($BiFeO_3$) deposited onto a semi-infinite transparent substrate ($SrTiO_3$). Note that the probe wavelength λ=587 nm corresponds to a quanta of energy smaller than both the band gaps of $SrTiO_3$ and $BiFeO_3$ justifying the transparency of this assembly. With that assumption, the transient optical reflectivity magnitude becomes $\Delta R/R$ (35-38):

$$\frac{\Delta R}{R} = \mathbb{R}\Bigg(2C\Bigg[-i\frac{\partial k_{\mathrm{BiFeO_3}}}{\partial \eta}\int_0^H \eta_{\mathrm{BiFeO_3}}(z,t)\left[r_{\mathrm{BiFeO_3-SrTiO_3}}e^{-ik_{\mathrm{BiFeO_3}}(H-z)}+e^{ik_{\mathrm{BiFeO_3}}(H-z)}\right]^2 dz - i\left(1-r^2_{\mathrm{BiFeO_3-SrTiO_3}}\right)\frac{\partial k_{\mathrm{SrTiO_3}}}{\partial \eta}\int_H^\infty \eta_{\mathrm{SrTiO_3}}(z,t)e^{i2k_{\mathrm{SrTiO_3}}z}dz\Bigg]\Bigg), \quad \text{(S4)}$$

with

$$C = \frac{\left(1-r^2_{air-\mathrm{BiFeO_3}}\right)}{\left(r_{air-\mathrm{BiFeO_3}}e^{ik_{\mathrm{BiFeO_3}}H}+r_{\mathrm{BiFeO_3-BiFeO_3}}e^{-ik_{\mathrm{BiFeO_3}}H}\right)} \cdot \frac{1}{\left(e^{ik_{\mathrm{BiFeO_3}}H}+r_{air-\mathrm{BiFeO_3}}r_{\mathrm{BiFeO_3-SrTiO_3}}e^{-ik_{\mathrm{BiFeO_3}}H}\right)}$$

$k_{\mathrm{BiFeO_3}} = 2\pi n_{\mathrm{BiFeO_3}}/\lambda$ and $k_{\mathrm{SrTiO_3}} = 2\pi n_{\mathrm{SrTiO_3}}/\lambda$ are the probe beam wavevector in the $BiFeO_3$ layer and the $SrTiO_3$ substrate, respectively. λ is the probe wavelength in the 181 vacuum, $\eta(z, t)$ is the acoustic strain, H is the thickness of the $BiFeO_3$ layer. The notation

$r_{\mathrm{BiFeO_3-SrTiO_3}}$ is used for the optical reflection coefficient at the interface between the media $BiFeO_3$ and $SrTiO_3$, for a normal incident probe beam:

$$r_{\mathrm{BiFeO_3-SrTiO_3}} = \frac{n_{\mathrm{BiFeO_3}}-n_{\mathrm{SrTiO_3}}}{n_{\mathrm{BiFeO_3}}+n_{\mathrm{SrTiO_3}}} \quad \text{(S5)}$$

We use a similar expression to calculate $r_{air-\mathrm{BiFeO_3}}$. The first integral term in Eq. S4 is the photoelastic contribution due to the scattering of the probe beam electric field by the acoustic strain $\eta(z, t)$ within the $BiFeO_3$ transparent layer, i.e. the so-called Brillouin scattering. The last integral term accounts for the photoelastic contribution of the substrate. Like the first integral term, it describes how the electric field of the light is scattered in the substrate by the acoustic strain. For a probe wavelength below the band gap of both $BiFeO_3$ and $SrTiO_3$, a narrowband acousto-optic detection (detection of Brillouin oscillations) is involved in our experiment (35), i.e. we detect only the Brillouin components as mentioned in the manuscript Methods. Note that in Eq. S4, the strain field $\eta(z, t)$ can be either the longitudinal and shear strain components. Cosnequently, the photoelastic coupling term $\frac{\partial k_{\mathrm{BiFeO_3}}}{\partial \eta}$ has to be adapted for the longitudinal (η_L) and shear (η_T) strains.

The simulation uses as adjustable parameters only the photoelastic coefficients $\frac{\partial k_{\mathrm{BiFeO_3}}}{\partial \eta}$ (for both the longitudinal and shear strain) and $\frac{\partial k_{\mathrm{SrTiO_3}}}{\partial \eta}$ which all are real in our case since we have a probe photon energy smaller than the $BiFeO_3$ and $SrTiO_3$ band gaps. The computed signal is shown in Figs. 3A-B in the main manuscript. We are able to reproduce the experimental signal with a good agreement.

IV. SUPPLEMENTARY NOTE 4. SELECTED BRAGG PLANES IN TIME-RESOLVED X-RAY DIFFRACTION MEASUREMENTS AND THEIR RELATION WITH THE STRAIN

A. Selected Bragg planes in time-resolved X-ray diffraction measurements

For the time-resolved X-ray diffraction experiment we selected two Bragg plane families being either sensitive $(403)_\mathrm{m}$ or not $(530)_\mathrm{m}$ to the shear strain. As shown in Fig. S6, when

the pump laser impinges on the sample (i.e., on the (b_{m},c_{m}) plane in the monoclinic frame), for symmetry reasons, the light-induced strain is composed of longitudinal (LA) and shear (TA) strain components. Since the plane (a_{m},c_{m}) is a mirror plane, the shear motion cannot be along b_{m} direction and is restricted to the $\mathrm{c_m}$ direction, i.e., this shear strain induces a sliding of the $(530)_{\mathrm{m}}$ and $(530)_{\mathrm{m}}$ planes. Consequently, there is no contribution of this shear component on the light-induced change of the interplanar distance of the $(530)_{\mathrm{m}}$ planes. On the opposite, this shear strain (atomic displacement along c_{m} direction) induces a modification of the interplanar distance that is different for $(403)_{\mathrm{m}}$ and $(40\bar{3})_{\mathrm{m}}$. In the next subsection B of this Supplementary Note IV, the relation between diffraction angle ω and strain component values are derived for each of the above-mentioned lattice planes.

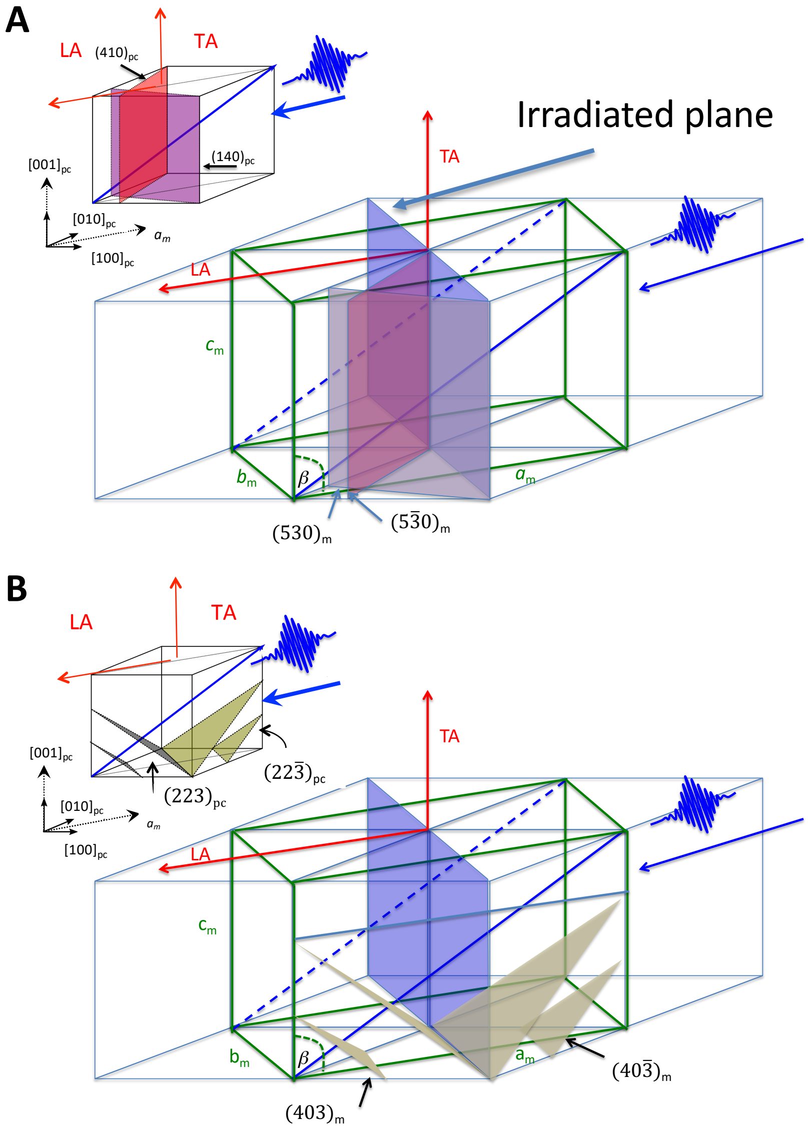

FIG. S6. **Representation of the (a) $(530)_m/(410)_{pc}$ and (b) $(403)_m/(223)_{pc}$ lattice planes** in the pseudo-cubic (small figures) and monoclinic frames (large figures). The subscripts m and pc refer to the monoclinic and to the pseudo-cubic unit cells, respectively.

B. Relation between the experimentally measured shift of ω angle and the light-induced strain components

In the figure S7, we present the geometry for the X-ray diffraction experiment based on a rocking curve (ω) scans. In the case of asymmetric Bragg planes (i.e. not parallel to the surface of the sample), when the light induces some longitudinal and shear strains (denoted η_L and η_T, respectively), there is not only a change of the interplanar distance ($\Delta d/d$), but also a change of the Bragg plane orientation with respect to the direction of the incident X-ray beam. This tilt angle is depicted as $\Delta\alpha$ in the Figure S7 and the latter one also depends on the strain components. As a consequence, the variation of the ω angle we have
is not only governed by the Bragg angle change ($\Delta\theta$) but also by this tilt angle $\Delta\alpha = \alpha' - \alpha$ (α' is the angle when the unit cell is submitted to the strain). Both contributions depend on the strain components following:

$$\omega' - \omega_0 = \Delta\theta(\eta_L, \eta_T) - \Delta\alpha(\eta_L, \eta_T) = -\frac{\Delta d(\eta_L, \eta_T)}{d} tan(\theta) - \Delta\alpha(\eta_L, \eta_T) \tag{S6}$$

where ω' and ω_0 denote the time-dependent diffraction angle, and the diffraction angle at equilibrium, respectively.

We consider first the case of (h0l) planes. Using the reasonable orthorhombic approximation (monoclinic angle β taken as 90 ° instead of 89.5 °), we can show first that the interplanar distance $\frac{\Delta d(\eta_L, \eta_T)}{d}$ can be written as function of the variation of the α angle with:

$$\begin{aligned} d &= \frac{a}{|l|}\sin(\alpha) \\ \Delta d &= \frac{a}{|l|}\Delta\alpha\cos(\alpha) \\ \frac{\Delta d}{d} &= \frac{\Delta\alpha}{\tan(\alpha)} = \frac{h}{\sqrt{2}|l|}\Delta\alpha \end{aligned} \tag{S7}$$

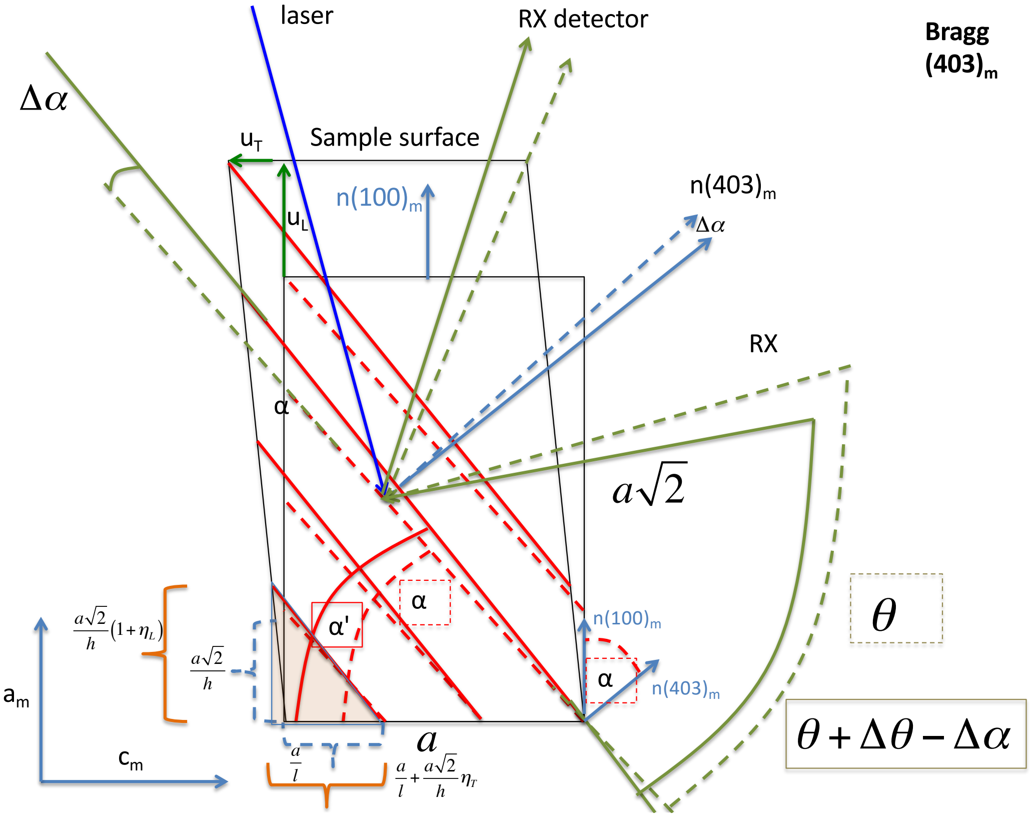

FIG. S7. **Sketch of the sample orientation** during the X-ray diffraction measurements on the 403_m reflection. $n(403)_\mathrm{m}$ stands for the vector normal to the $(403)_\mathrm{m}$ planes. $\mathrm{u_L}$ and $\mathrm{u_T}$ are the out-of-plane and in-plane variations of the lattice parameters

We have also the two following expressions for $\alpha' = \alpha + \Delta\alpha$ and α:

$$\sin(\alpha + \Delta\alpha) = \frac{\sqrt{2}(1+\eta_L)}{\sqrt{(\frac{h}{|l|} - \frac{l}{|l|}\sqrt{2}\eta_T)^2 + 2(1+\eta_L)^2}} \tag{S8}$$

and

$$\sin(\alpha) = \frac{\sqrt{2}}{\sqrt{(\frac{h}{|l|})^2 + 2}} \tag{S9}$$

With the trigonometric relation $\sin(\alpha+\Delta\alpha) = \sin(\alpha)\cos(\Delta\alpha) + \cos(\alpha)\sin(\Delta\alpha)$ and con-

sidering small variation of the α angle ($\Delta\alpha \ll 1$), and the corresponding Taylor developments $\cos(\Delta\alpha) \sim 1$ and $\sin(\Delta\alpha) \sim \Delta\alpha$, we arrive to:

$$\begin{aligned}\Delta\alpha &= [\sin(\alpha+\Delta\alpha) - \sin(\alpha)]/\cos(\alpha) \\ &= \left[\frac{\sqrt{2}(1+\eta_L)}{\sqrt{(\frac{h}{|l|} - \frac{l}{|l|}\sqrt{2}\eta_T)^2 + 2(1+\eta_L)^2}} - \frac{\sqrt{2}}{\sqrt{(\frac{h}{|l|})^2 + 2}}\right] / \cos(\alpha)\end{aligned} \tag{S10}$$

with further Taylor developments, we arrive to:

$$\Delta\alpha = \sqrt{2}\left(2 + \frac{h^2}{|l|}\right)^{-1}\left[\frac{h}{|l|}\eta_L + \sqrt{2}\frac{l}{|l|}\eta_T\right] \tag{S11}$$

With Eq. S7 and Eq. S6, Eq. S11 is rewritten as:

$$\Delta\omega(\eta_L, \eta_T) = \omega' - \omega_0 = -A(\theta, h, l)\left[\eta_L\frac{h}{|l|} + \sqrt{2}\frac{l}{|l|}\eta_T\right] \tag{S12}$$

with

$$A(\theta, h, |l|) = \left(\tan(\theta)\frac{h}{|l|} + \sqrt{2}\right)\left(2 + \frac{h^2}{|l|}\right)^{-1} \tag{S13}$$

A similar calculation can be realized for the (hk0) planes. We have first:

$$\begin{aligned} d &= \frac{a\sqrt{2}}{|k|}\sin(\alpha) \\ \Delta d &= \frac{a\sqrt{2}}{|k|}\Delta\alpha\cos(\alpha) \\ \frac{\Delta d}{d} &= \frac{\Delta\alpha}{\tan(\alpha)} = \frac{h}{|k|}\Delta\alpha \end{aligned} \tag{S14}$$

and

$$\sin(\alpha + \Delta\alpha) = \frac{(1+\eta_L)}{\sqrt{(1+\eta_L)^2 + \frac{h^2}{|k^2|}}} \tag{S15}$$

with

$$\cos(\alpha) = \frac{1}{\sqrt{1 + \frac{|k^2|}{h^2}}} \tag{S16}$$

We finally arrive to:

$$\Delta\omega(\eta_L, \eta_T) = \omega' - \omega_0 = -B(\theta, h, |k|)\eta_L \tag{S17}$$

with

$$B(\theta, h, |k|) = (\tan(\theta)\frac{h}{|k|} + 1)\frac{h|k|}{h^2 + |k|^2} \tag{S18}$$

For numerical applications, we have considered for the pseudo-cubic approximation that α=30 ° and α =45.6 ° for $5\pm30_{\mathrm{m}}$ and $40\pm3_{\mathrm{m}}$ reflections, respectively. Note that the experimental Bragg angles are θ= 64.690 °, 64.931 ° and 67.175 ° for $(5\pm30)_{\mathrm{m}}$, $(403)_{\mathrm{m}}$ and $(403)_{\overline{\mathrm{m}}}$ respectively.

V. SUPPLEMENTARY NOTE 5. FURTHER DETAILS ON THE TIME-RESOLVED X-RAY DIFFRACTION EXPERIMENTS PERFORMED AT THE CRISTAL BEAM-LINE AT THE SOLEIL SYNCHROTRON, FRANCE

A. 2D images of Bragg peaks

Figure S8 shows images of the 403_{m} Bragg peak as detected before (left) and at a time of 200 ps after laser excitation (right). The analysis of the 2D images obtained by ω scans reveals that the diffraction takes place within the incident plane of the X-ray beam, indicating that the light-induced atomic motion takes place in the $(a_{\mathrm{m}}, c_{\mathrm{m}})$ plane consistently with the

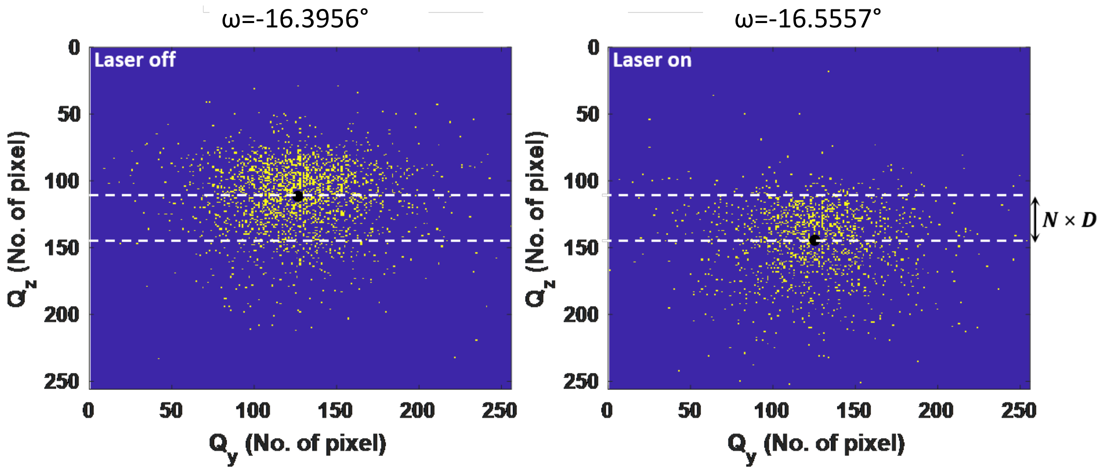

FIG. S8. **Diffraction images of the 403_{m} Bragg peak, taken 200 ps before (left) and 200 ps after (right) light pulse excitation**, at an ω angle corresponding to the maximum diffracted intensity. The black dots depict the barycenter of the Bragg peak. The quantity $N \times D$ represents the shift of the Bragg peak where N in the number of pixels and D the size of a pixel of the detector.

B. Renormalized fluence on the sample

To evaluate the fluence during the measurement of the $40\overline{3}_{\mathrm{m}}$ and $5\overline{3}0_{\mathrm{m}}$ Bragg peaks, one must account for a different sample orientation. In the experiment, the Bragg angles of both these families of lattice planes are nearly equal ($\theta_{h0l_{\mathrm{m}}} \sim \theta_{hk0_{\mathrm{m}}}$, Fig. S9). Therefore, since $(403)_{\mathrm{m}}$ and $(530)_{\mathrm{m}}$ planes have different inclination regarding to the normal of the free surface, the sample must be rotated differently to measure the corresponding reflections. The variation is $|\alpha_{403_{\mathrm{m}}} - \alpha_{530_{\mathrm{m}}}| = 16$ °, which corresponds to the aforementioned relative orientation of the normals of the planes $(403)_{\mathrm{m}}$ and $(530)_{\mathrm{m}}$ relative to the normal of the sample (c_{m}), see also Fig. S7. Experimentally, this leads to pump laser fluences of 5.2 and 4.2 mJ.cm^{-2} during the measurement of 403_{m} and 530_{m} reflections, respectively. This correction factor is considered when we compare the amplitude of the lattice strain deduced from the study of $(403)_{\mathrm{m}}$ and $(530)_{\mathrm{m}}$ planes as shown in Fig. 6A. The fluence has been calculated with a footprint of the pump beam estimated with the FWHM of the pump gaussian beam.

We note that the effect of the pump light polarization is negligible. Considering the values of the refractive index available in the literature only for the rhombohedral $BiFeO_3$ material, we have calculated that pumping with an electric field along either the ordinary or extraordinary axe leads to a respective optical absorption of 69% and 71%. Similar conclusions were already discussed in the case of an excitation of a $BiFeO_3$ single crystal having a $3m$ point group symmetry (11).

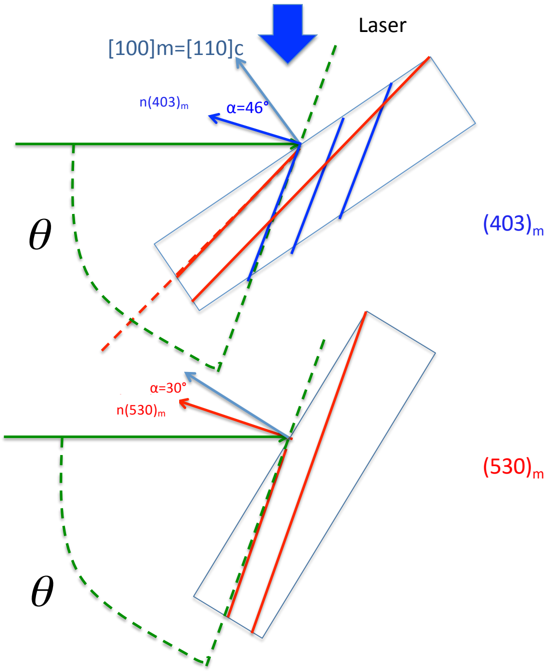

FIG. S9. **Sketch of the sample orientation** during the X-ray diffraction experiments with $(403)_\mathrm{m}$ and $(530)_\mathrm{m}$ lattice planes.

REFERENCES

1. M. E. Lines, A. M. Glass, Principles and Applications of Ferroelectrics and Related Materials. 1977.

2. M. Fiebig, T. Lottermoser, D. Meier, M. Trassin, The evolution of multiferroics. *Nat. Rev. Mater.* **1**, 16046 (2016).

3. N Spaldin, R Ramesh, Advances in magnetoelectric multiferroics. *Nat. Mater.* **18**, 203–212, (2019).

4. Y. Zhang, J. Dai, X. Zhong, D. Zhang, G. Zhong, J. Li, Probing ultrafast dynamics of ferroelectrics by time-resolved pump-probe spectroscopy. *Adv. Sci.* **8**, e2102488, (2021).

5. T. Linker, K.-I. Nomura, A. Aditya, S. Fukshima, R. K. Kalia, A. Krishnamoorthy, A. Nakano, P. Rajak, K. Shimmura, F. Shimojo, P. Vashishta, Exploring far-from-equilibrium ultrafast polarization control in ferroelectric oxides with excited-state neural network quantum molecular dynamics. *Sci. Adv.* **8**, eabk2625 (2022).

6. T. F. Nova, A. S. Disa, M. Fechner, A. Cavalleri, Metastable ferroelectricity in optically strained $SrTiO_3$. *Science* **364**, 1075–1079 (2019).

7. X. Li, T. Qiu, J. Zhang, E. Baldini, J. Lu, A. M. Rappe, K. A. Nelson, Terahertz field induced ferroelectricity in quantum paraelectric $SrTiO_3$. *Science* **364**, 1079–1082 (2019).

8. R. Mankowsky, A. von Hoegen, M. First, A. Cavalleri, Ultrafast reversal of the ferroelectric polarization. *Phys. Rev. Lett.* **118**, 197601 (2017).

9. H. Wen, P. Chen, M. P. Cosgriff, D. A. Walko, J. H. Lee, C. Adamo, R. D. Schaller, J. F. Ihlefeld, E. M. Dufresne, D. G. Schlom, P. G. Evans, J. W. Freeland, Y. Li, Electronic origin of ultrafast photoinduced strain in $BiFeO_3$. *Phys. Rev. Lett.* **110**, 037601 (2013).

10. D. Schick, M. Herzog, H. Wen, P. Chen, C. Adamo, P. Gaal, D. G. Schlom, P. G. Evans, Y. Li, M. Bargheer, Localized excited charge carriers generate ultrafast inhomogeneous strain in the multiferroic $BiFeO_3$. *Phys. Rev. Lett.* **112**, 097602 (2014).

11. V. Juvé, R. Gu, S. Gable, T. Maroutian, G. Vaudel, S. Matzen, N. Chigarev, S. Raetz, V. E. Gusev, M. Viret, A. Jarnac, C. Laulhé, A. A. Maznev, B. Dkhil, P. Ruello, Ultrafast light-induced shear strain probed by time-resolved x-ray diffraction: Multiferroic $BiFeO_3$ as a case study. *Phys. Rev. B* **102**, 220303 (2020).

12. D. Daranciang, M. J. Highland, H. Wen, S. M. Young, N. C. Brandt, H. Y. Hwang, M. Vattilana, M. Nicoul, F. Quirin, J. Goodfellow, T. Qi, I. Grinberg, D. M. Fritz, Marco Cammarata, D. Zhu, H. T. Lemke, D. A. Walko, E. M. Dufresne, Y. Li, J. Larsson, D. A. Reis, K. Sokolowski-Tinten, K. A. Nelson, A. M. Rappe, P. H. Fuoss, G. B. Stephenson, A. M. Lindenberg, Ultrafast photovoltaic response in ferroelectric nanolayers. *Phys. Rev. Lett.* **108**, 087601 (2012).

13. C. Paillard, B. Xu, B. Dkhil, G. Geneste, L. Bellaiche, Photostriction in ferroelectrics from density functional theory. *Phys. Rev. Lett.* **116**, 247401 (2016).

14. S. Matzen, L. Guillemot, T. Maroutian, S. K. K. Patel, H. Wen, A. D. Dihiara, G. Agnus, O. G. Shpyrko, E. E. Fullerton, D. Ravelosona, P. Lecoeur, R. Kukreja, Tuning ultrafast photoinduced strain in ferroelectric-based devices. *Adv. Electron. Mater.* **5**, 1800709 (2019).

15. H. J. Lee, Y. Ahn, S. D. Marks, D. S. Gyan, E. C. Landahl, J. Y. Lee, T. Y. Kim, S. Unithrattil, S. H. Chun, S. Kim, S.-Y. Park, C. I. Eom, A. H. Wen, D. G. Schlom, S. Lee, J. Y. Jo, P. G. Evans, Subpicosecond optical stress generation in multiferroic $bifeo_3$. *Nano Lett.* **22**, 4294–4300 (2022).

16. C. K. Schmising, M. Bargheer, M. Kiel, N. Zhavoronkov, M. Woerner, T. Elsaesser, I. Vrejoiu, D. Hesse, M. Alexe, Coupled ultrafast lattice and polarization dynamics in ferroelectric nanolayers. *Phys. Rev. Lett.* **98**, 257601 (2007).

17. P. Ruello, T. Pézeril, S. Avanesyan, G. Vaudel, V. Gusev, I. C. Infante, B. Dkhil, Photoexcitation of gigahertz longitudinal and shear acoustic waves in $BiFeO_3$ multiferroic single crystal. *Appli. Phys. Lett.* **100**, 212906 (2012).

18. M. Lejman, G. Vaudel, I. C. Infante, P. Gemeiner, V. E. Gusev, B. Dkhil, P. Ruello, Giant ultrafast photo-induced shear strain in ferroelectric $BiFeO_3$. *Nat. Commun.* **5**, 4301 (2014).

19. M. Lejman, G. Vaudel, I. C. Infante, I. Chaban, T. Pézeril, M. Edely, G. F. Nataf, M. Guennou, J. Kreisel, V. E. Gusev, B. Dkhil, P. Ruello, Ultrafast acousto-optic mode conversion in optically birefringent ferroelectrics. *Nat. Commun.* **7**, 12345 (2016).

20. L. Y. Chen, J. C. Yang, C. W. Luo, C. W. Laing, K. H. Wu, J.-Y. Lin, T. M. Uen, J. Y. Juang, Y. H. Chu, T. Kobayashi. Ultrafast photoinduced mechanical strain in epitaxial $BiFeO_3$ thin films. *Appl. Phys. Lett.* **101**, 041902 (2012).

21. P. Khan, M. Kanamaru, K. Matsumoto, T. Ito, T. Satoh, Ultrafast light-driven simultaneous excitation of coherent terahertz magnons and phonons in multiferroic $BiFeO_3$. *Phys. Rev. B* **101**, 134413 (2020).

22. P. Chen, C. Paillard, H. J. Zhao, J. Íñiguez, L. Bellaiche, Deterministic control of ferroelectric polarization by ultrafast laser pulses *Nat. Commun.* **13**, 1–8 (2022).

23. A.V. Kimel, A.M. Kalashnikova, A. Pogrebna, A.K. Zvezdin, Fundamentals and perspectives of ultrafast photoferroic recording. *Phys. Rep.* **852**, 1–46 (2020).

24. J.-Y. Chauleau, T. Chirac, S. Fusil, V. Garcia, W. Akhtar, J. Tranchida, P. Thibaudeau, I. Gross, C. Blouzon, A. Finco, M. Bibes, B. Dkhil, D. D. Khalyavin, P. Manuel, V. Jacques, N. Jaouen, and M. Viret, Electric and antiferromagnetic chiral textures at multiferroic domain walls. *Nat. Mater.* **19**, 386–390 (2020).

25. A. Haykal, J. Fischer, W. Akhtar, J.-Y. Chauleau, D. Sando, A. Finco, F. Godel, Y. A. Birkholzer, C. Carretero, N. Jaouen, M. Bibes, M. Viret, S. Fusil, V. Jacques, V. Garcia, Antiferromagnetic textures in $BiFeO_3$ controlled by strain and electric field. *Nat. Commun.* **11**, 1704 (2020).

26. D. Sando, F. Appert, Bin Xu, O. Paull, S. R. Burns, C. Carretero, B. Dupe, V. Garcia, Y. Gallais, A. Sacuto, M. Cazayous, B. Dkhil, J. M. Le Breton, A. Barthelemy, M. Bibes, L. Bellaiche, V. Nagarajan, J. Juraszek, A magnetic phase diagram for nanoscale epitaxial $BiFeO_3$ films. *Appl. Phys. Rev.* **6**, 041404 (2019).

27. D. Sando, Strain and orientation engineering in ABO_3 perovskite oxide thin films. *J. Phys. Condens. Matter* **34**, 153001 (2022).

28. H. J. Lee, Y. Ahn, S. D. Marks, E. C. Landahl, S. Zhuang, M. H. Yusuf, M. Dawber, J. Y. Lee, T. Y. Kim, S. Unithrattil, S. H. Chun, S. Kim, I. Eom, S.-Y. Park, K. S. Kim, S. Lee, J. Y. Jo, J. Hu, P. G. Evans, Structural evidence for ultrafast polarization rotation in ferroelectric/dielectric superlattice nanodomains. *Phys. Rev. X* **11**, 031031 (2021).

29. Y. Zhang, Y. Tan, D. Sando, L.-Q. Chen, N. Valanoor, Y. Zhu, M.-G. Han, Controlled nucleation and stabilization of ferroelectric domain wall patterns in epitaxial (110) bismuth ferrite heterostructures. *Adv. Funct. Mater.* **30**, 2003571 (2020).

30. D. Sando, T. Young, R. Bulanadi, X. Cheng, Y. Zhou, M. Weyland, P. Munroe, V. Nagarajan, Designer defect stabilization of the super tetragonal phase in >70-nm-thick $BiFeO_3$ films on $LaAlO_3$ substrates. *Jap. J. Appli. Phys.* **57**, 0902B2 (2018).

31. S. G. Choi, H. T. Yi, S.-W. Cheong, J. N. Hilfiker, R. France, A. G. Norman, Optical anisotropy and charge-transfer transition energies in BiFeO3from 1.0 to 5.5 ev. *Phys. Rev. B* **83**, 100101 (2011).

32. C. Thomsen, H. T. Grahn, H. J. Maris, J. Tauc, Surface generation and detection of phonons by picosecond light pulses. *Phys. Rev. B* **34**, 4129–4138 (1986).

33. P. Ruello, V. E. Gusev, Physical mechanisms of coherent acoustic phonons generation by ultrafast laser action. *Ultrasonics* **56**, 21–35 (2015).

34. M. Lejman, C. Paillard, V. Juvé, G. Vaudel, N. Guiblin, L. Bellaiche, M. Viret, V. E. Gusev, B. Dkhil, P. Ruello, Magnetoelastic and magnetoelectric couplings across the antiferromagnetic transition in multiferroic $BiFeO_3$. *Phys. Rev. B* **99**, 104103 (2019).

35. O. B. Wright, Thickness and sound velocity measurement in thin transparent films with laser picosecond acoustics. *J. Appl. Phys.* **71**, 1617–1629 (1992).

36. V. E. Gusev, Laser hypersonics in fundamental and applied research. *Acustic-acta acustica* **82**, S37–S45 1996.

37. O. B. Wright, O. Matsuda, Laser picosecond acoustics with oblique probe light incidence. *Rev. Sci. Instr.* **74**, 895–897 (2003).

38. C. Mechri, P. Ruello, V. Gusev, Confined coherent acoustic modes in a tubular nanoporous alumina film probed by picosecond acoustics methods. *New J. Phys.* **14**, 023048 (2012).

39. D. Mounier, P. Picart, P. Babilotte, P. Ruello, J.-M. Breteau, T. Pézeril, G. Vaudel, M. Kouyaté, V. Gusev, Jones matrix formalism for the theory of picosecond shear acoustic pulse detection. *Optic Express*, **18**, 6767–6778, (2010).

40. D Schick, A Bojahr, R Shayduk M Herzog, C von Korff Schmising, M Bargheer, udkm1Dsim—A simulation toolkit for 1d ultrafast dynamics in condensed matter. *Comput. Phys. Commun.* **185**, 651–660 (2014).

41. V. E. Gusev, P. Ruello, Advances in applications of time-domain brillouin scattering for nanoscale imaging. *Appl. Phys. Rev.*, **5**, 031101 (2018).

42. D. A. Reis, M. F. DeCamp, P. H. Bucksbaum, R. Clarke, E. Dufresne, M. Hertlein, R. Merlin, R. Falcone, H. Kapteyn, M. M. Murnane, J. Larsson, Th. Missalla, J. S. Wark, Probing impulsive strain propagation with x-ray pulses. *Phys. Rev. Lett.* **86**, 3072–3075 (2001).

43. T. Pézeril, P. Ruello, S. Gougeon, N. Chigarev, D. Mounier, J.-M. Breteau, P. Picart, V. Gusev. Generation and detection of plane coherent shear picosecond acoustic pulses by lasers: Experiment and theory. *Phys. Rev. B* **75**, 174307 (2007).

44. D. Schick, M. Herzog, A. Bojahr, W. Leitenberger, A. Hertwig, R. Shayduk, M. Bargheer, Ultrafast lattice response of photoexcited thin films studied by x-ray diffraction. *Struct. Dyn.* **1**, 064501 (2014).

45. I. Ponomareva, L. Bellaiche, Nature of dynamical coupling between polarization and strain in nanoscale ferroelectrics from first principles. *Phys. Rev. Lett.* **101**, 107602 (2008).

46. H. Huyan, L. Li, C. Addiego, W. Gao, X. Pan, Structures and electronic properties of domain walls in $BiFeO_3$ thin films. *Natl. Sci. Rev.* **6**, 669–683 (2019).

47. A. S. Everhardt, S. Matzen, N. Domingo, G. Catalan, B. Noheda, Ferroelectric domain structures in low-strain $BaTiO_3$. *Adv. Electron. Mater.* **2**, 1500214 (2016).

48. E. Gradauskaite, P. Meisenheimer, M. Muller, J. Heron, M. Trassin, Multiferroic heterostructures for spintronics. *Phys. Sci. Rev.* **6**, 0072 (2021).

49. L. W. Martin, A. M. Rappe, Thin-film ferroelectric materials and their applications. *Nat. Rev. Mater.* **2**, 16087 (2017).

50. D. Sando, M. Han, V. Govinden, O. Paull, F. Appert, C. Carrétéro, J. Fischer, A. Barthélémy, M. Bibes, V. Garcia, S. Fusil, B. Dkhil, J. Juraszek, Y. Zhu, X. Ma, V. Nagarajan, Interfacial Strain Gradients Control Nanoscale Domain Morphology in Epitaxial $BiFeO_3$ Multiferroic Films. *Adv. Funct. Mater.* **30**, 2000343 (2020).

51. A. Kumar, R. C. Rai, N. J. Podraza, S. Denev, M. Ramirez, Y.-H. Chu, L. W. Martin, J. Ihlefeld, T. Heeg, J. Schubert, Darrell G. Schlom, J. Orenstein, R. Ramesh, Robert W. Collins, Janice L. Musfeldt, V. Gopalan, Linear and nonlinear optical properties of $BiFeO_3$. *Appl. Phys. Lett.* **92**, 121915 (2008).

52. B. Gu, Y. Wang, J. Wang, W. Ji, Femtosecond third-order optical nonlinearity of $BiFeO_3$. *Optics Expr.* **17**, 10970 (2009).

53. D. Sando, P. Hermet, J. Allibe, J. Bourderionnet, S. Fusil, C. Carrétero, E. Jacquet, J.-C. Mage, D. Dolfi, A. Barthélemy, P. Ghosez, M. Bibes, Linear electro-optic effect in multiferroic $BiFeO_3$ thin films. *Phys. Rev. B* **89** 195106 (2014).

54. M.-A. Tordeux, J. Barros, A. Bence, P. Brunelle, N. Hubert, M. Labat, A. Nadji, L. Nadolski, P. Lebasque, J.-P. Pollina, C. Evain, Low-alpha operation for the soleil storage ring, in *Proceedings of the 2012 International Particle Accelerator Conference* (IPAC, 2012), pp. 1608.

55. D. Bachiller-Perea, Y.-M. Abiven, J. Bisou, P. Fertey, P. Grybos, A. Jarnac, B. Kanouté, A. Koziol, F. Langlois, C. Laulhé, F. Legrand, P. Maj, C. Menneglier, A. Noureddine, F. Orsini, G. Thibaux, A. Dawiec, First pump–probe–probe hard x-ray diffraction experiments with a 2d hybrid pixel detector developed at the SOLEIL synchrotron. *J. Synchrotron Radiat.* **27**, 340–350 (2020).

56. S. L. Shang, G. Sheng, Y. Wang, L. Q. Chen, Z. K. Liu, Elastic properties of cubic and rhombohedral $BiFeO_3$ from first-principles calculations. *Phys. Rev. B* **80**, 052102 (2009).

57. E. Borissenko, M. Goffinet, A. Bosak, P. Rovillain, M. Cazayous, D. Colson, P. Ghosez, M. Krisch, Lattice dynamics of multiferroic $BiFeO_3$ studied by inelastic x-ray scattering. *J. Phys. Condens. Matter* **25**, 102201 (2013).

58. P. Hemme, P. Djemia, P. Rovillain, Y. Gallais, A. Sacuto, A. Forget, D. Colson, E. Charron, B. Perrin, L. Belliard, M. Cazayous, Elastic properties assessment in the multiferroic $BiFeO_3$ by pump and probe method. *Appl. Phys. Lett*. **118**, 062902 (2021).

59. L. Maerten, A. Bojahr, M. Gohlke, M. Rössle, M. Bargheer, Coupling of GHz phonons to ferroelastic domain walls in $SrTiO_3$. *Phys. Rev. Lett*. **114**, 047401 (2015).

60. S. Yamanaka, T. Maekawa, H. Muta, T. Matsuda, S. Kobayashi, K. Kurosaki, Thermophysical properties of $SrHfO_3$ and $SrRuO_3$. *J. Solid State Chem.* **177**, 3484–3489 (2004).

61. R. O. Bell, G. Rupprecht, Elastic constants of strontium titanate. *Phys. Rev.* **129**, 90 (1963).
